# Targeting mitochondrial translation and OXPHOS in high-grade serous ovarian carcinoma eliminates stem-like cells

**DOI:** 10.1038/s41419-025-07987-1

**Published:** 2025-10-06

**Authors:** Aravindan Narayanan, Souvik Guha, Avinash Mali, Sharmila A. Bapat

**Affiliations:** 1 BRIC-National Centre for Cell Science, Savitribai Phule Pune University, Pune, India; 2 Present Address: Centre for Interdisciplinary Research in Basic Sciences, Jamia Millia Islamia, New Delhi, India

**Keywords:** Ovarian cancer, Cancer stem cells, Cancer metabolism

## Abstract

Ex vivo stem cell self-renewal and maintenance is supported by absence of serum-derived mitogens. In the present study, we sought to elucidate the proteomes of stem-like cells grown in serum-free media across a panel of high-grade serous ovarian cancer cell lines, which encompass a gradient from epithelial, intermediate and mesenchymal cell phenotypes to recapitulate the heterogeneity of the disease. MaxQuant-based label-free quantification of proteins identified that despite their different cellular and molecular architectures, all phenotypes exhibited mitochondria- and stemness-related pathways under conditions of serum starvation, although the specific proteins involved were discrete to each phenotype. This suggests that common cellular programs in a disease can be mediated through variable biological networks that generates molecular heterogeneity. We further explored if these pathways are inter-related, co-regulated or just incidentally associated in response to an environment depleted of growth factors and mitogens. Irrespective of their phenotype, cell lines on serum-starvation displayed an increased amount of mitochondrial DNA, mitochondrial biogenesis and mitochondrial activity with a switch from glycolysis to oxidative phosphorylation fuelled by the fatty acid oxidation. Ultra-structural studies implicated this metabolic fluctuation was regulated by dynamic mitochondrial remodelling. This also led us to explore a possible therapeutic strategy of targeting mitochondrial function to restrict tumor regenerative potential and disease recurrence. Conclusively, these new avenues contribute to a more comprehensive understanding of ovarian cancer.

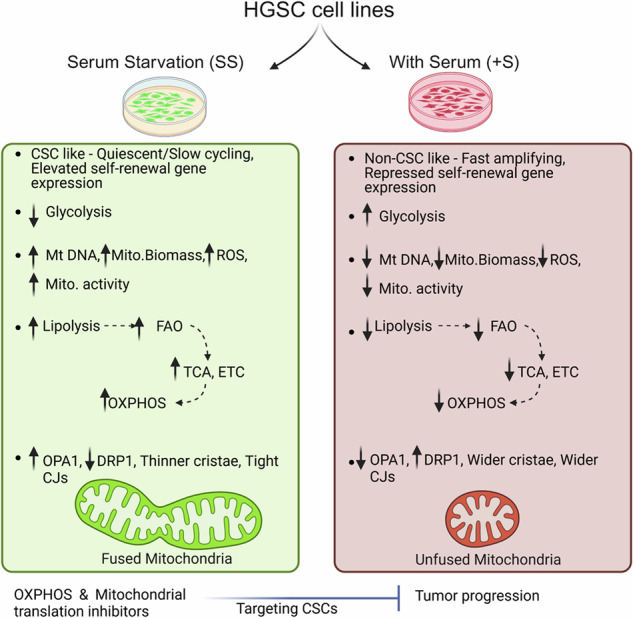

## Introduction

Amongst gynecological malignancies, high-grade serous ovarian carcinoma (HGSC) is regarded as most aggressive and presents challenges of early detection and treatment emerging from the complex biologies of inter- and intra-tumor heterogeneity [1–4]. Amongst the several attempts towards addressing these issues, we had earlier resolved inter-tumor molecular heterogeneity into three discrete molecular subtypes [5–7]. While these clinically validated out in patient tumors, we also studied their correlation with distinct cellular phenotypes in vitro that facilitated deeper molecular investigations [8, 9]. Briefly, the three tumor subtypes encompass five cell phenotypes, each of which was exemplified by at least one cell line. Each of these epithelial (E-OVCAR3), intermediate epithelial (iE-CAOV3), epithelial-mesenchymal hybrid (E/M-OVCA420), intermediate mesenchymal (iM-A4), and mesenchymal (M-OVMZ6) states displays a distinct molecular and phenotypical architecture as well as biological functions.

Resolution of intra-tumor heterogeneity reveals yet another pivotal aspect of tumor biology encompassing genomic changes (mutations, aneuploidy) and cellular dynamics involving intrinsic dormancy (quiescence) and regeneration by cancer stem cells (CSCs) within tumors that generate hierarchies of multiple clones [10]. CSCs are implicated in processes such as metastasis, drug resistance and recurrent disease by leveraging their state of quiescence, presence of drug efflux pumps, signaling pathways that aid self-renewal, such as Hedgehog signaling pathway, Notch signaling pathway and Wnt signaling [11, 12]. Studying CSCs in vitro in the presence of serum that contributes several essential growth factors and mitogens perturbs their quiescence, creates a state of rapid proliferation rather than that of a tightly self-renewal program. Such system artifacts make it irrelevant to applying outcomes to the in situ state where cells do not divide continuously. Serum depletion has hence, earlier been indicated to provide for more relevant systems of CSC maintenance by permitting entry into a state of quiescence or that of slow cycling that also influences cellular plasticity, migration and invasiveness of tumor cells [13–17]. While serum depletion eliminates cellular replicative stress, it may impose nutritive stress and altered cellular energetics that remains to be elucidated.

In the present study, we initially affirmed our working hypothesis that serum starvation (SS) would provide an in-situ relevance for studying the molecular networks contributing to CSC maintenance and/or self-renewal. Following this, differential proteomics across the spectrum of clinical heterogeneity of HGSC cell subtypes identified several enriched proteins under SS conditions, none of which were common across all the phenotypes yet, mitochondrial translation and activation were indicated to be a common function. More specifically, mitochondrial metabolic pathways *viz*. TCA and OXPHOS involving mitochondrial matrix and Electron transport chain (ETC) proteins were prominently enhanced in all phenotypes under SS, along with acquisition of a stem-like state in association with upregulation of mitochondrial proteins. Our exploration of the same suggests a coregulation and cross-talk between the two pathways. This vulnerability may present a potential opportunity to target residual regenerative potential despite the inherent heterogeneity of HGSC.

## Results

### Serum starvation modulates enrichment of ‘stemness’ features and a phenotype-specific proteomic profile in HGSC cell lines

**Fig. 1:**
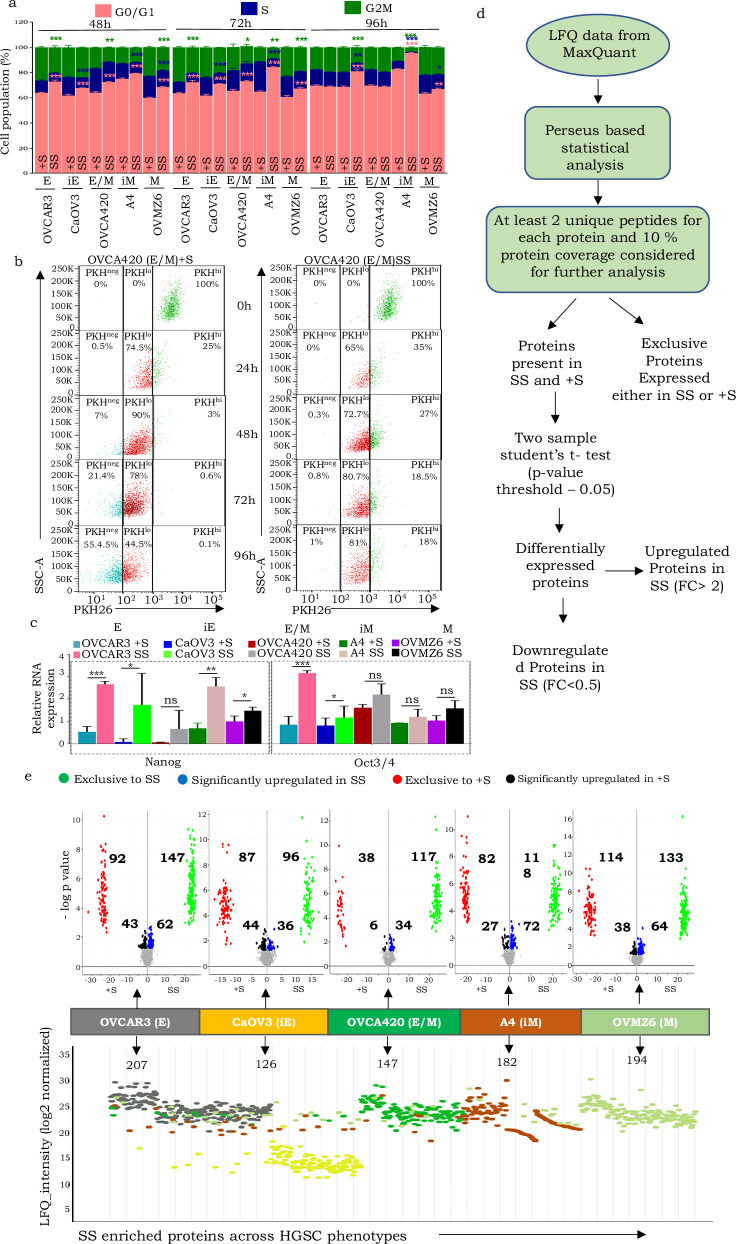
Serum starvation enriches slow cycling stem-like cells across a phenotype gradient ranging from epithelial (E-OVCAR3), intermediate epithelial (iE-CaOV3), epithelial-mesenchymal hybrid (E/M-OVCA420), intermediate mesenchymal (iM- A4) and mesenchymal (M-OVMZ6) in HGSC cells with discrete enriched (exclusive and upregulated) proteomic profiles associated with each phenotypic state. **a** Altered propidium iodide (PI)-based cell cycle kinetics following SS of HGSC cell lines across the phenotypic gradient across three time points (48 h, 72 h and 96 h). Within each cluster of stacked-bar graphs, the first one represents data from serum-supplemented (+S) conditions, while the subsequent bar depicts corresponding values under serum-free (SS) conditions. Colored asterisks on the second bar of each pair (SS condition) indicate statistically significant differences (p-values) in each cell cycle phase relative to their respective +S controls; **b** Representative flow cytometry-based dot-plot of PKHhi, PKHlo and PKHneg fractions in OVCA420 (E/M) + S vs SS sample, left and right panel respectively, at different time points (0 h, 24 h, 48 h, 72 h and 96 h) under SS and +S conditions across the phenotype gradient revealed through PKH label-chase (green-PKHhi, red-PKHlo, blue-PKHneg); **c** Relative RNA expression of self-renewal genes (Nanog,Oct3/4), in SS and +S states across different phenotypes; **d** Analytical pipeline for label-free quantification and identification of differentially expressed proteins; **e** Differentially expressed protein candidates across the phenotypic gradient under SS & +S conditions, Top panel – Volcano plots (red–exclusive to +S, black–upregulated in +S, green-exclusive to SS, blue– upregulated in SS), Lower panel - LFQ_intensity plot (gray–E, yellow–iE, green–E/M, brown-iM, light green–M) *p < 0.05, **p < 0.01, and ***p < 0.001.

Comparing cycling profiles of HGSC cell lines under conditions of serum starvation (SS) in comparison with their growth in the presence of serum (+S; controls) indicated significant enrichment of a G0/G1 fraction and reduced S phase across the entire gradient of phenotypes in HGSC cell lines (Fig. 1a, Supplementary Fig. 1a). We assessed the slow-cycling and quiescent nature of this fraction using a vital fluorophore PKH26, that binds to the plasma membrane and is progressively quenched with each cell division [18]. This allowed us to distinguish HGSC cell populations in vitro into distinct PKH fractions based on label retention, *viz*. PKH^hi^, PKH^lo^, and PKH^neg^ [18]. The PKH^hi^ fraction represents quiescent/slow-cycling cells, PKH^lo^ includes cells that have undergone limited divisions, and PKH^neg^ reflects rapidly dividing cells that undergo complete label quenching. All phenotypes retained a substantial proportion of cells in the PKH^hi^ and PKH^lo^ fractions at all time points examined post serum starvation. In contrast, in presence of serum these fractions are rapidly depleted (Fig. 1b; Supplementary Fig. 1b). Notably, an increased number of quiescent/slow-cycling cells under serum-depletion was also associated with significantly enhanced expression of the self-renewal marker Nanog across all phenotypes examined (E/M state being an outlier), while Oct4 appeared to be enriched in the epithelial phenotypes (Fig. 1c; Supplementary Fig. 1d). These findings suggest that serum starvation may serve as an effective surrogate model for studying the stem-like state in HGSC across its molecular phenotypes.

We further developed a pipeline for mass spectrometry-based protein profiling following 48 h of SS using MaxQuant^TM^-based label-free quantification (LFQ) and data analysis (Fig. 1d). This identified differentially enriched, significantly upregulated proteins exclusive to each of the two groups within each HGSC phenotype (SS and +S), as well as across the entire gradient of phenotypes (Supplementary Fig. 1e, f; Supplementary Table 1). Proteins that were either exclusive to or significantly upregulated (>2-fold change) in individual groups (+S or SS) were considered as “enriched proteins” for further analysis (Supplementary Table 1). Interestingly, no proteins were commonly enriched in all phenotypes under SS conditions, although neighboring states in the gradient shared a few candidates (Supplementary Fig. 1f). Conclusively, the absence of serum-derived growth factors and mitogens that perhaps could lead genes towards acquisition of a ‘stem-like’ state under conditions of serum depletion state in HGSC as affirmed through lowered cell cycle kinetics and discreet enrichment of self-renewing cells within the population associated with a specific yet varied protein expression in each phenotype (Fig. 1e). Such discrete proteomic profiles despite a common stem cell state across phenotypes is well aligned with our earlier studies correlating phenotype-specific molecular networks and responses to microenvironmental cues [7, 9].

### Mitochondrial metabolic pathways are enriched under conditions of serum deprivation regardless of cell phenotype

**Fig. 2:**
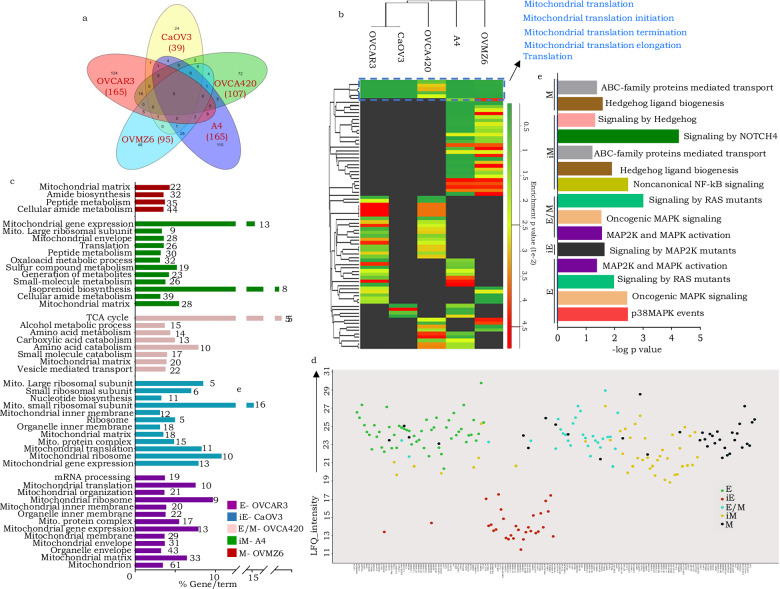
Activation of mitochondrial machinery is common across all phenotypes on serum depletion. **a** Venn diagram representing enriched (shared and unique) pathways following SS; **b** A heatmap illustrating RAECTOME pathway enrichment, based on enrichment *p*-values, depicting molecular pathways significantly enriched in SS compared to the corresponding +S condition across the phenotypic spectrum (enriched mitochondrial translation pathways, p < 0.05, are highlighted at the top of the heatmap); **c** Bar graph indicating enriched GO terms across HGSC phenotypes following SS (number of proteins corresponding to each GO term indicated on the bar); **d** LFQ intensity scatter plot indicating distribution of SS-enriched mitochondria-related proteins across the phenotype gradient (green-E, red-iE, blue-E/M, yellow-iM and black-M); **e** Horizontal bar graph representing enrichment of stemness-related pathways under SS across phenotypes *p < 0.05, **p < 0.01, and ***p < 0.001.

Phenotype-specific functions were further delineated through pathway analysis of enriched proteins (exclusive and significantly upregulated_>2-fold change) in each phenotype following serum depletion. The E, iE and E/M phenotypes were thus revealed as being quite discrete through their association with differential biological functions and pathways, while M and iM phenotypes shared several common pathways (Fig. 2a, b). The most interesting and unexpectedly common enriched pathway across all phenotypes was of mitochondrial translation (initiation, elongation, termination - REACTOME and DAVID analyses) associated with enrichment of Tricarboxylic acid (TCA) cycle, ETC, oxidoreductase complex, mitochondrial ribosomes, mitochondrial membrane, mitochondrial gene expression, mitochondrial matrix etc. (Gene Ontology analysis, GO; ClueGo v2.5.10; Fig. 2c; Supplementary Fig. 2). This association was unique and emerged despite a majority of mitochondria-associated proteins being distinctly unique to each phenotype and limited commonality of proteins between neighboring phenotypes (Fig. 2d). This could reflect on the nature and stability of discrete associated molecular networks governing its phenotype and may also extend to a distinct cellular architecture in each tumor class [8]. Concurrent enrichment of CSC-related molecular pathways was also evident across the phenotypes affirming recapitulation of stemness features in response to SS through similar responses between neighboring phenotypes (MAPK signaling and RAS mutants enriched in E, iE and E/M phenotype whereas Hedgehog signaling, ABC-family proteins mediated transport etc. were enriched in iM and M; Fig. 2e). Conclusively, these data suggesting activation of mitochondrial metabolic pathways following serum deprivation regardless of the unique protein profiles across phenotypes may be pivotal as a stress response, which is associated with acquisition of a stem-like state, and are consistent with previous reports associating stemness with cellular stress [19–23].

### Serum starvation triggers a metabolic switch from glycolysis to OXPHOS, with increased mitochondrial biogenesis, DNA copy number, membrane potential and ROS

**Fig. 3:**
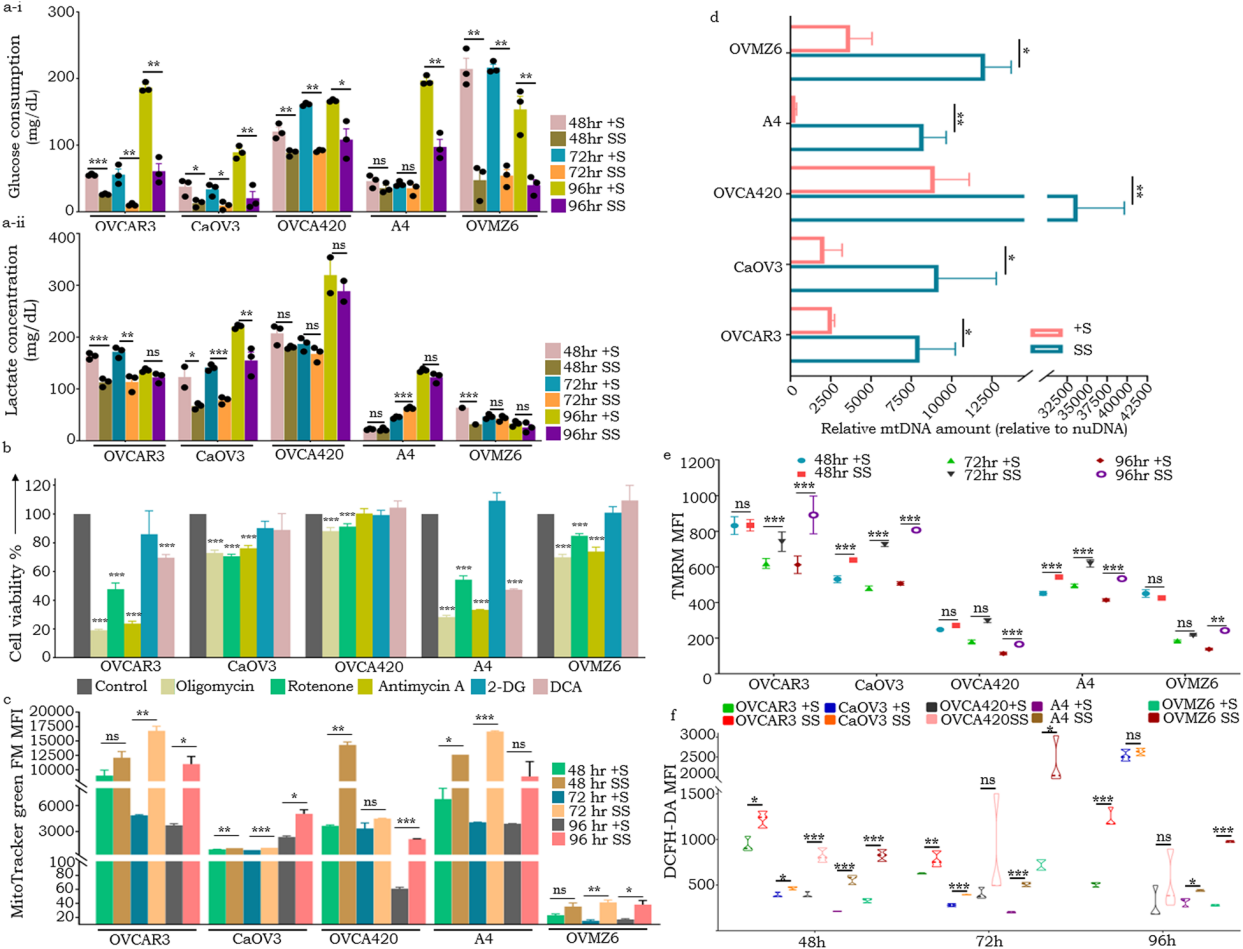
HGSC cells tide over SS by virtue of enhanced mitochondrial activity and preferential switch towards OXPHOS as a source of energy. **a-i**, **a-ii**. Glucose consumption and lactate assay respectively across all phenotypes under SS & +S conditions (rosy beige-48h +S, golden olive-48h SS, teal blue-72h +S, coral orange-72h SS, pear green-96h +S, purple-96h SS); **b** Representative cell viability across all phenotypes following treatment with IC50 concentrations of OXPHOS (pale goldenrod-Oligomycin,13-22 uM; teal green-Rotenone,11-22 uM; pear green-AntimycinA,11-40 uM) and glycolysis inhibitors (teal blue-2-DG,150-340 uM; rosy beige-DCA,19–32 uM; black-untreated controls) for each cell line following serum-deprivation for 48h (cell-line specific IC50 values are given in Supplementary Fig. 3b); **c** Representative mitochondrial mass analysis (MitoTracker green_FM assay) under SS & +S conditions; **d** Relative mtDNA content under 48h SS & +S conditions across cell phenotypes; **e** Representative mitochondrial membrane potential (TMRM assay) under SS & +S conditions across phenotypes and at different time points; **f** Truncated violin plot indicating mitochondria-associated ROS levels in under SS & +S conditions across phenotypes *p < 0.05, **p < 0.01, and ***p < 0.001.

The most widely studied effects of mitochondria in cancer relate to tumor cell metabolism, with glycolysis being a preferred pathway [24]. Surprisingly, we observed that SS contrarily led to reduced glucose uptake and lactate production across all phenotypes, suggesting a preferred switch towards oxidative phosphorylation (OXPHOS; Fig. 3a–i, a–ii). Inhibition of glycolysis using 2-DG and sodium dichloroacetate (DCA), or of OXPHOS employing Rotenone, Antimycin A, and Oligomycin, revealed that only OXPHOS inhibition significantly compromised cell viability under serum-deprived conditions across the various phenotypic subtypes. An exception was noted with DCA, which elicited a marked reduction in survival compared to controls specifically in the E (OVCAR3) and iM (A4) phenotypes. This differential response is likely attributable to the mechanistic effect of DCA. DCA primarily inhibits pyruvate dehydrogenase kinase (PDK), thereby activating pyruvate dehydrogenase (PDH) and promoting mitochondrial respiration [25]. Consequently, we hypothesize that the survival of a subset HGSC phenotypes was affected by DCA through its modulation of mitochondrial metabolic flux. Additionally, the unique metabolomic architectures intrinsic to each phenotypic subtype contributed to the heterogeneity in response, rendering the effects of DCA non-universal across all phenotypes (Fig. 3b; Supplementary Fig. 3a-i, b). We further explored and affirmed through flow cytometry that such enrichment of mitochondrial molecular signatures on serum withdrawal is associated with increased mitochondrial biomass across all phenotypes and at all the timepoints examined (Fig. 3c, Supplementary Fig. 3c; upper panel). Along with this, increased mtDNA copy number (as compared to nuclear DNA) and mitochondrial membrane potential (TMRM assay) indicating activation of mitochondria on SS were noted (Fig. 3d, e, Supplementary Fig. 3c-lower panel), concurrently with increased levels of reactive oxygen species (ROS), which may not be cytotoxic as the HGSC cells have entered a quiescent state (Fig. 3f, Supplementary Fig. 3-middle panel). These data indicate that, unlike rapidly dividing cancer cells that rely on glycolysis, quiescent CSCs have more active mitochondria and rely on OXPHOS as their primary source of energy, which enables them to survive under conditions of nutritional stress.

### HGSC cells subjected to serum starvation rely on stored fatty acids for their energy requirements

**Fig. 4:**
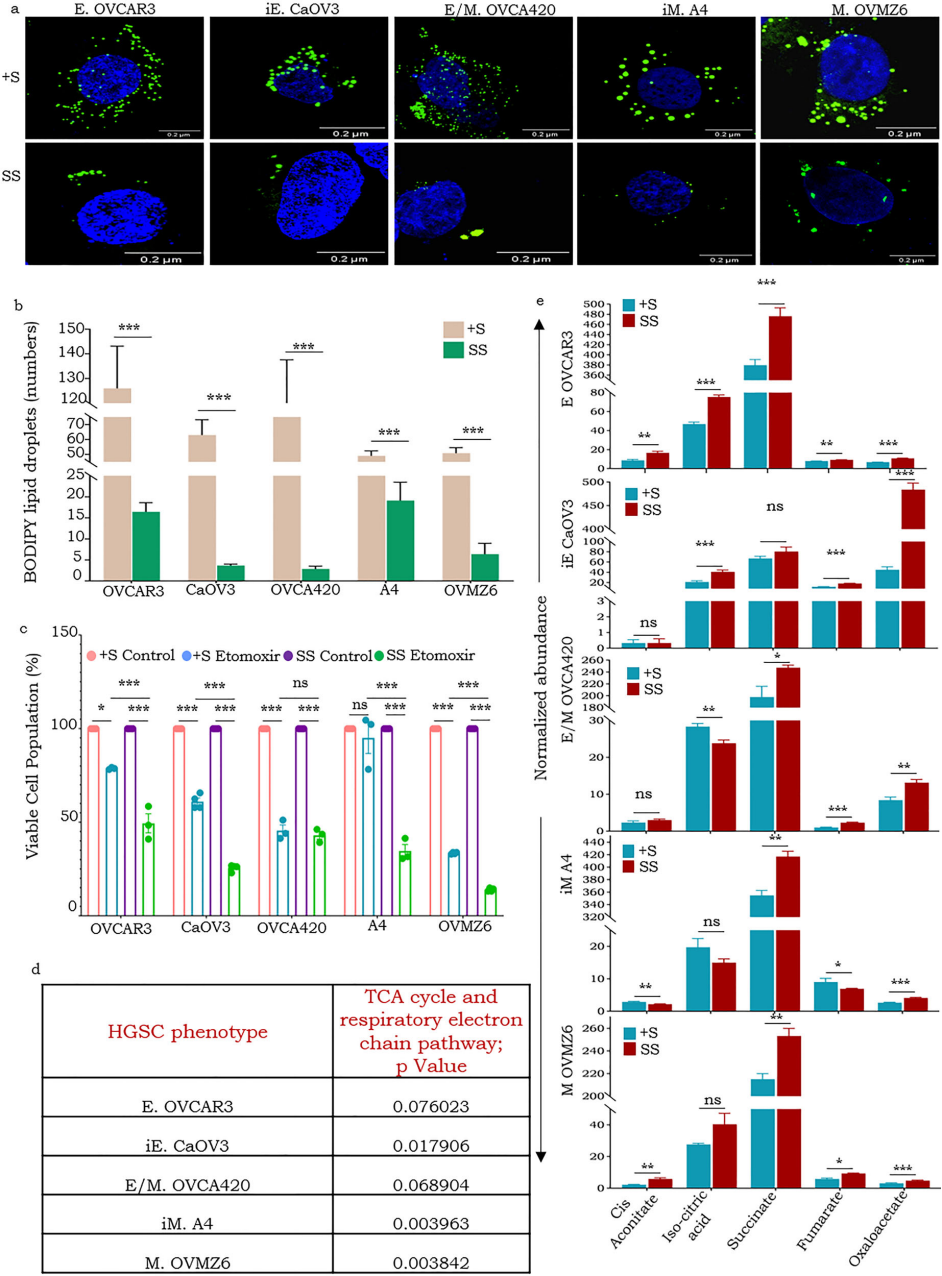
HGSC cells rely on fatty acid oxidation and fatty acids to fuel the TCA cycle and respiratory ETC for their energy needs under SS conditions. **a** Representative confocal images identifying BODIPY lipid droplets (green) in cells under SS and +S conditions (lower and upper panel respectively), nuclei stained with Hoechst; **b** Quantification of lipid droplets (Confocal imaging- 50 cells from each sample) across HGSC phenotypes under SS and +S conditions; **c** Void bar-graph indicating cell viability under SS and +S conditions following exposure to Etomoxir; **d** Table indicating significant enrichment of the TCA cycle and electron transport chain across HGSC phenotypes; **e** Abundance of TCA metabolites under SS and +S conditions across the gradient from E to M phenotypes (top to bottom) *p < 0.05, **p < 0.01, and ***p < 0.001.

To explore the possibility that cells under serum starvation may rely on stored fatty acids (FAs), through fatty acid oxidation (FAO), as a source of energy, we profiled the frequency of lipid droplets (LDs) in cells under SS for 48 h as compared with controls (+S). A substantial decrease in LDs following starvation was a common feature across all phenotypes (Fig. 4a, b), indicating stored FAs mobilization for energy generation under nutrient deprivation. However, the precise mechanisms underlying this mobilization, whether it is predominantly mediated by lipophagy or conventional lipolysis, is remain to be elucidated. The dependence on FAO and use of LD-derived FAs as a source of energy was further affirmed by profiling cell survival on exposure to Etomoxir (an inhibitor of carnitine palmitoyltransferase 1, CPT1). This clearly displayed all cell lines except OVCA420 (E/M phenotype) under SS conditions to be more sensitive to the inhibitor over controls (Fig. 4c). The comparable sensitivity of OVCA420 to Etomoxir under both conditions is likely to be due to some undetermined phenotype-specific metabolic feature that leads to elevated basal level FAs transport to mitochondria and fatty acid β-oxidation activity. Since pathway analysis had earlier also indicated significant enrichment of “TCA cycle and respiratory electron transport” across all phenotypes (Fig. 4d), we profiled and identified enhanced production of TCA metabolites (Cis-Aconitate, Iso-citric acid, Succinate, Fumarate and Oxaloacetate) after 48 h of SS as compared with their respective +S controls (Fig. 4e, Supplementary Fig. 4). Conclusively, HGSC cells on serum depletion to rely on FAO and stored FAs to fuel TCA cycle and ETC and subsequently OXPHOS for their energy requirements.

### Mitochondria in HGSC cells under conditions of serum starvation undergo fusion and display thinner cristae and tight crista junctions

**Fig. 5:**
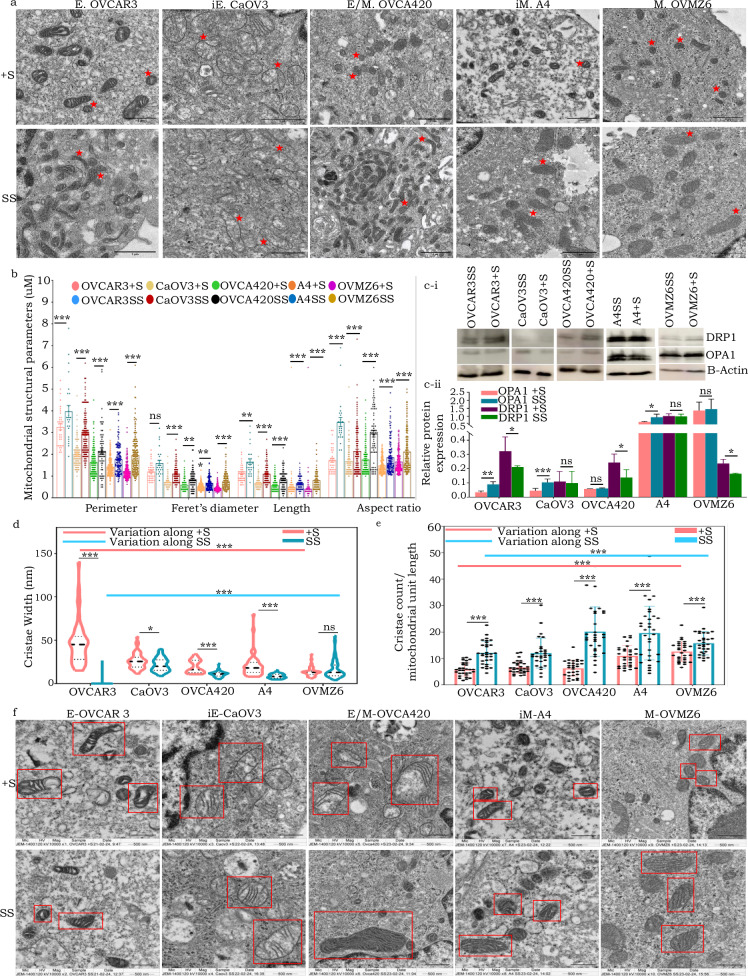
SS-driven mitochondrial fusion is associated with thinner cristae, tight CJs and an increased number of cristae per unit mitochondrial length. **a** Representative 2D-TEM images highlighting mitochondria (red asterisk) in HGSC cells under 48 h of SS in comparison with controls; **b** Bar-graph representation indicating quantitative mitochondrial structural parameters from 2D-TEM images (a minimum of 30 mitochondria from 12 cells were analyzed each in +S and SS condition across the different phenotypes); **c-i** Representative Western blots indicating expression of mitochondrial fusion and fission proteins (OPA1 and DRP1 respectively) 48 h SS *vs* + S samples, actin used as loading controls; **c-ii** Quantification of OPA1 and DRP1 protein expression in Western blots; **d** Truncated violin plot representing cristae width computed from 2D-TEM images (a minimum of 50 mitochondria from 12 cells were analyzed each in +S and SS condition across the differenst phenotypes); **e** Representative bar-graph wherein individual data points indicate number of cristae per unit mitochondrial length across phenotype gradient under SS and +S conditions; **f** Representative TEM images indicating the mitochondria and cristae (highlighted by red squares) under SS and +S conditions, lower and upper panels respectively across the phenotypic gradient *p < 0.05, **p < 0.01, and ***p < 0.001.

In alignment with the principle that “form follows function,” we hypothesized that altered metabolism of HGSC cells in response to serum deprivation may also be associated with changes in mitochondrial morphology. Indeed, a shift from a globular to more elongated presentation of mitochondria across all the HGSC phenotypes was identified, quantitation of which affirmed the same [higher Aspect Ratio (AR) and Form Factor with lowered Roundness and Solidity features in 48-h SS cells compared +S controls; Supplementary Fig. 5a-i, a-ii]. Validation through two-dimensional Transmission Electron Microscopy (2D-TEM) imaging and analysis further suggested potential mitochondrial fusion events in HGSC SS cells as (marked increase in Feret’s diameter, AR, perimeter and length; Fig. 5a, b). This was further supported by the protein level expression of the mitochondrial dynamics regulators. An overall trend of increased OPA1 expression and decreased DRP1 levels was observed in HGSC phenotypes under serum-deprived conditions; with CaOV3 (iE) and A4 (iM) cells being outliers for DRP1 expression, while OVCA420 (E/M) and OVMZ6 (M) were outliers for OPA1 expression (Fig. 5c–i, c–ii). This differential response may reflect heterogeneity in mitochondrial dynamics across different HGSC phenotypes, potentially mediated by interactions with other regulatory proteins.

Given that cristae play a crucial role in OXPHOS by housing ETC complexes and facilitating ATP synthesis through H+ gradient optimization, we examined their structural dynamics under conditions of serum deprivation. 2D-TEM images of cells revealed an inverse correlation between cristae width and number of cristae per unit mitochondrial length (Fig. 5d, e). Interestingly, the (E) state was associated with fewer and broader cristae that increased progressively towards the (M) state under +S conditions. A reversal of this trend was observed on SS, along with significant reduction in width of crista junctions (CJs) and increased number of cristae per unit length of mitochondria as compared with controls across all phenotypes (Fig. 5d–f; Supplementary Fig. 5b-i, b-ii). Collectively, these observations strongly demonstrate that HGSC cells adapt to serum deprivation through a metabolic switch to OXPHOS and remodeling of their mitochondrial ultrastructural features to optimize energy demands.

### Mathematical modeling supports mitochondrial dynamics readouts and predicts higher ATP turn over in fused mitochondria as a response to serum starvation

**Fig. 6:**
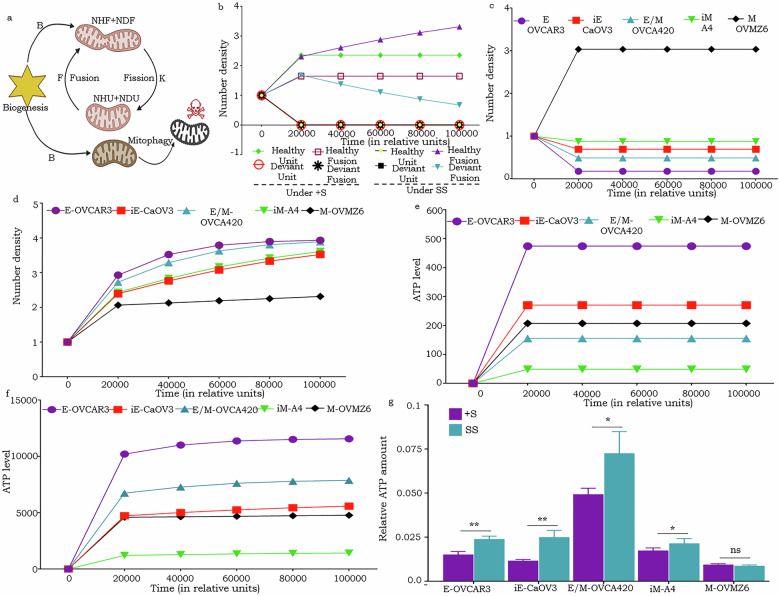
Mathematical modeling of mitochondrial dynamics and energetics of HGSC phenotypes under SS and normal conditions. **a** Graphical representation of mitochondrial dynamics based on parameters described in the text; **b** Line plot indicating the number of HF mitochondria over time in stress free (+S) and SS conditions; Line plot indicating the number of mitochondria over time in different HGSC phenotypes under +S (**c**) and SS (**d**); **e**, **f** Line plot indicating the ATP energetics across the phenotypes under +S and SS conditions, respectively; **g** Bar-graph indicating levels of ATP production in SS and +S conditions *p < 0.05, **p < 0.01, and ***p < 0.001.

Towards a deeper understanding and prediction of phenotype-dependent mitochondrial dynamics and energy outcomes in response to SS (considered as nutritional stress in the system), we modeled some of the ultrastructural features based on the following assumptions (Fig. 6a; Table 1)—

- Mitochondrial number is a function of mitochondrial biogenesis and mitophagy;
- A system possesses healthy mitochondria (those less likely to undergo mtDNA mutations) as well as deviant derivatives (prone to mtDNA mutations).
- Healthy and deviant mitochondria have identical rates of fusion and fission that are reflected in the expression levels of OPA1 and DRP1 (key mitochondrial fusion and fission proteins respectively);
- Four classes of mitochondria *viz*. healthy units (HU), healthy fused (HF), deviant units (DU) and deviant fused (DF) were considered; since both deviant and healthy mitochondria have the same rate of biogenesis, hence formation rate (B) of all four classes is uniform;
- Healthy and deviant mitochondria contribute to ATP production; however, the former are more efficient, producing ATP at a factor (α) greater than the latter;
- Fused mitochondria exhibit significantly enhanced ATP production efficiency, modeled as a fused efficiency factor (ϵ). This indicates that fused mitochondria (both healthy and deviant) are more productive than their unfused counterparts;
- The rate of mitochondrial ATP production (θ) is based on mitochondrial structural parameters including, number of cristae per unit length (c_n), membrane potential [e^(m_p)], average cristae width (c_w), and cristae junction width (c_jw);
- As ATP machinery resides within cristae, greater number of cristae correlates with increased ATP production;
- ATP production is directly proportional to membrane potential; higher membrane potential hence indicates increased ATP synthesis;
- Cristae junctions are crucial for minimizing proton leakage from the inner cristae space, and hence increased numbers enhance proton density and ATP production;
- Thinner cristae also contribute to increased proton density and improved OXPHOS efficiency;
- Healthy as well as deviant mitochondria initially display increased rate of fusion in response to serum starvation stress to mitigate mitophagy; concurrently, the rate of fission is expected to decrease under stress. Hence, our model incorporates an exponential increase in initial fusion (F_0) and decrease in fission (K_0) rates;
- The processes of mitochondrial fusion and fission are factored into the model as being influenced by the availability and concentration of ATP.

**Table 1:** Table indicating the mitochondrial parameters considered for mathematical modeling.

	Cristae Width (c_w)	Cristae count per unit length (c_n)	Cristae Junction Width	Membrane Potential (m_p)	Fusion Promoter (OPA1)	Fission Promoter (DRP1)
OVCAR3 + S	1	0.28	0.62	0.99	0.02	0.31
OVCAR3SS	0.05	0.6	0.3	1	0.06	0.2
CAOV3 + S	0.55	0.32	1	0.63	0.03	0.1
CAOV3SS	0.47	0.59	0.73	0.76	0.07	0.09
OVCA420 + S	0.42	0.31	0.89	0.29	0.03	0.23
OVCA420SS	0.23	1	0.47	0.32	0.04	0.13
A4 + S	0.44	0.54	0.63	0.54	0.44	1
A4SS	0.18	0.97	0.34	0.65	0.65	0.98
OVMZ6 + S	0.29	0.62	0.41	0.54	0.93	0.23
OVMZ6SS	0.34	0.78	0.23	0.51	1	0.16

The above assumptions were applied to the derivation of coupled ordinary differential equations as follows:

$$\frac{d{N}_{{HU}}}{{dt}}=\left(B-{M}_{H}-F\left[{ATP}\right]{e}^{{stress}}\right){N}_{{HU}}+K\left[{ATP}\right]{e}^{-{stress}}{N}_{{HF}}$$

$$\frac{d{N}_{{DU}}}{{dt}}=\left(B-{M}_{D}-F\left[{ATP}\right]{e}^{{stress}}\right){N}_{{DU}}+K\left[{ATP}\right]{e}^{-{stress}}{N}_{{DF}}$$

$$\frac{d{N}_{{HF}}}{{dt}}=\left(B-K\left[{ATP}\right]{e}^{-{stress}}\right){N}_{{HF}}+F\left[{ATP}\right]{e}^{{stress}}{N}_{{HU}}$$

$$\frac{d{N}_{{DF}}}{{dt}}=\left(B-K\left[{ATP}\right]{e}^{-{stress}}\right){N}_{{DF}}+F\left[{ATP}\right]{e}^{{stress}}{N}_{{DU}}$$

$$\frac{d\left[{ATP}\right]}{{dt}}=\theta \left[\epsilon \left(\alpha {N}_{{HF}}+{N}_{{DF}}\right)+\left({\alpha N}_{{HU}}+{N}_{{DU}}\right)\right]-\,\mu -K\left[{ATP}\right]\left({N}_{{HF}}+{N}_{{DF}}\right){e}^{-{stress}}-\,F\left[{ATP}\right]\left({N}_{{DU}}\right){e}^{{stress}}-F\left[{ATP}\right]\left({N}_{{HU}}\right){e}^{{stress}}$$

wherein,

(i) ATP production (*θ*) is assumed to be a function of all the above-mentioned parameters,
  $$\theta =\frac{{c}_{n}+{e}^{{m}_{p}}}{{c}_{w}+{c}_{{jw}}}$$
(ii) Mitochondrial fusion (*F*) and fission (*K*) rates are assumed to be a function of OPA1 and DRP1,
  $$F={F}_{0}* \left[{OPA}1\right]$$
  $$K={K}_{0}* \left[{DRP}1\right]$$
(iii) The rate of mitochondria created by biogenesis is equal to the total number of mitochondria that eliminated through mitophagy,
  $$B\left({N}_{{HU}}+{N}_{{HF}}+{N}_{{DU}}+{N}_{{DF}}\right)={M}_{H}{N}_{{HU}}+{M}_{D}{N}_{{DU}}$$
(iv) The initial conditions were set at,

$${N}_{{HU}}=1,{N}_{{HF}}=1,{N}_{{DU}}=1,{N}_{{DF}}=1,\left[{ATP}\right]=0,\mu =3$$

Our model thus predicted an increase in the number of HF mitochondria under serum stress, with HU and DU being almost completely depleted from the system over the course of time (Fig. 6b). This was substantiated experimentally through TEM analysis wherein regardless of the phenotype, the frequency of elongated HF mitochondria under SS was higher over that in a stress-free +S environment that displays higher frequency of HU mitochondria (Fig. 6b). In addressing the time-dependent mitochondrial dynamics of individual HGSC phenotypes, a spectrum of HF distribution was revealed ranging from being highest in “M” phenotype (OVMZ6) and lowest in the “E” phenotype (OVCAR3) under +S conditions; this was completely reversed on SS (Fig. 6c, d). Further prediction of cellular energetics revealed a remarkable increase in ATP production across all HGSC phenotypes following SS as compared with +S conditions (Fig. 6e, f). Within this, our model predicts E and E/M phenotypes (OVCAR3 and OVCA420 respectively) to be associated with maximal ATP production, while iE (A4) is likely to be the least energetic (Fig. 6f). Importantly, these predictions regarding cellular energetics were experimentally substantiated by assaying ATP, which corroborated the E, E/M and iE phenotypes to exhibit elevated levels of ATP production following SS (Fig. 6g).

### Inhibition of OXPHOS and mitochondrial translation impairs self-renewal in vitro and tumor progression in vivo

**Fig. 7:**
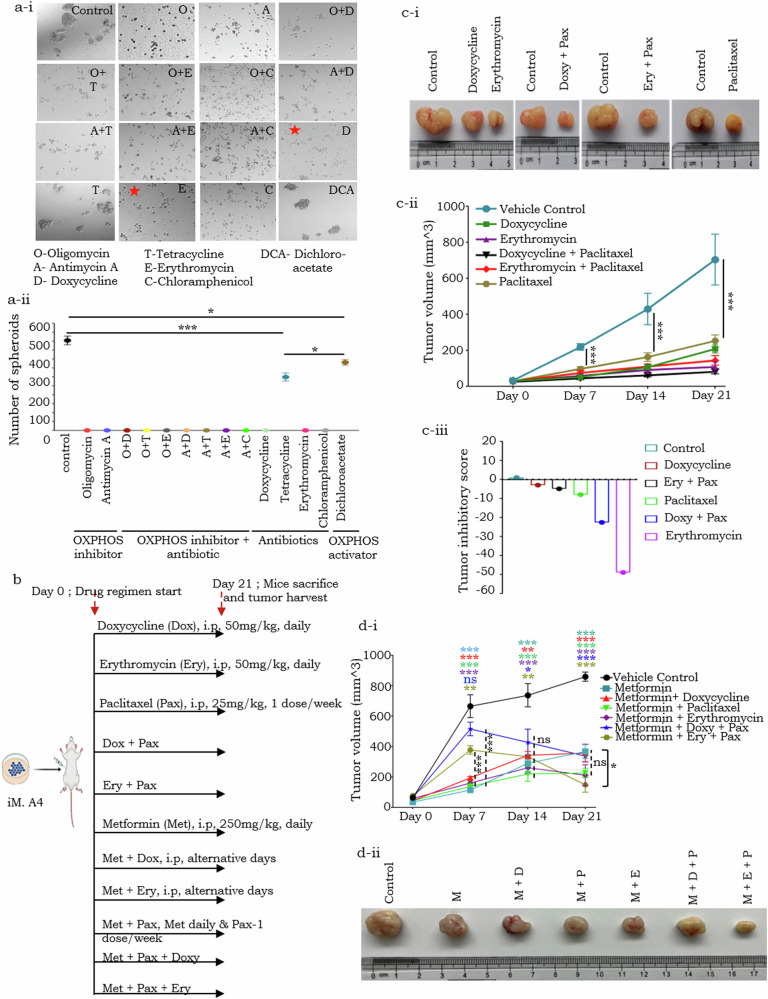
Inhibition of OXPHOS and mitochondrial translation significantly impairs spheroid formation in vitro and tumor progression in vivo. **a**–**i** Representative images and quantification of A4 spheroids exposed to various drugs and their combinations (Panels with red star denotes the antibiotic drugs Doxycycline and Erythromycin that most significantly inhibited spheroid formation over other the drugs, and hence were subsequently used for in vivo evaluation); **a-ii** Dot plot representing the number of spheroids under different drug treatment measured on Day14 (post cell seeding); **b** Schematic representation of drug regimen used in the study; **c-i** Representative A4 tumors harvested on Day 21 of different drug regimens; **c-ii** Line graph indicating A4 tumor volumes measured on Day 0,7,14 and 21 during treatment of various drugs (single and in combination; Day 0 corresponds to the initiation of treatment, which occurs 14 days post subcutaneous tumor cell inoculation); c-iii. Bar-graph indicating the anti-tumor efficacy of various drug combinations, Y-axis represents the tumor inhibitory score (where control represent a score of “1”); **d-i** Line graph depicting A4 tumor volumes over the course of treatment with the ETC complex I inhibitor metformin, administered either as a monotherapy or in combination with other therapeutic agents. Day 0 marks the initiation of treatment. Asterisks of different colors denote p-value significance (student’s *t* test) between each treatment group and the vehicle control on the corresponding day. The dotted line denotes *p*-vlaue significance among the various drug regimens on Days 7, 14, and 21, as assessed by one-way ANOVA. The solid line indicates the statistical significance of the comparison between Metformin monotherapy and the combination therapy comprising Metformin, Erythromycin, and Paclitaxel (Metformin + Ery + Pax); **d-ii** Representative A4 tumors harvested on 21st day after different drug regimens. *p < 0.05, **p < 0.01, and ***p < 0.001.

We further evaluated in vitro suspended spheroid-formation capability of A4 cells to validate the purported enhanced generation of stem-like cells following SS and their dependency on OXPHOS and mitochondrial functions for self-renewal and/or maintenance. Notably, a significant reduction in spheroid-forming capabilities was observed following individual and combined drug treatments, including OXPHOS (ETC) inhibitors and antibiotics (leveraging the similarity between bacterial and mitochondrial ribosomes that can inhibit mitochondrial translation; Supplementary Fig. 3a-i, b-i, b-ii; Supplementary Fig. 6a, b). While Doxycycline and Erythromycin exhibited the most pronounced inhibitory effects, Antimycin A, Oligomycin and Chloramphenicol also were inhibitory (Fig. 7a–i, a–ii). In contrast, Tetracycline induced spheroid formation, albeit the extent of formation was significantly reduced compared to the controls (Fig. 7a–i, a–ii). To assess whether enhanced OXPHOS could restore the spheroid-forming capacity of A4 cells compared to its inhibition, we treated the cells with DCA (augment OXPHOS by inhibiting PDK and subsequent activation of PDH). Notably, DCA treatment resulted in partial restoration of spheroid formation ability (Fig. 7a–i, a–ii). However, this restored capacity remained significantly below that observed in vehicle-treated control ability (Fig. 7a–i, a–ii). These findings suggest that the intrinsic balance between glycolysis and OXPHOS in untreated cells likely represents an optimal metabolic state for spheroid formation [26]. The modest increase in mitochondrial activity induced by DCA appears insufficient to surpass this physiological equilibrium (Fig. 7a–i, a–ii). Collectively, the data support a critical role for mitochondrial function in maintaining the self-renewal and stem-like properties of cancer cells.

We further explored if the association between mitochondrial activity and CSC self-renewal could be harnessed in situ using Doxycycline and Erythromycin (that exhibited the most pronounced inhibitory effects on spheroid formation). Indeed, the growth and progression of A4 xenografts in mice subjected to the antibiotic regimen, either alone or in conjunction with Paclitaxel were significantly reduced following treatment; despite the finding that some extent of drug resistance/evasion may emerge with Doxycycline treatments after Day 14 (Fig. 7b, c-i, c-ii, c-iii). A consequent label-chase in xenografts using the vital dye (PKH26) revealed an increase in the frequency of label retaining cells, however with significantly reduced tumor volumes following drug treatment as compared with vehicle controls. To quantify the efficacy of each treatment regimen, we calculated a “tumor inhibitory score” wherein label-retaining PKHhi fractions representing CSCs were normalized to tumor volumes under different drug regimens and compared with that of vehicle control (Methods). An enhanced tumor inhibitory score reflects on effective reduction in the number of slow-cycling/quiescent tumor cells following treatments (Fig. 1ci, cii, ciii; Supplementary Fig. 7a). Since CSCs may rely on OXPHOS, we also incorporated the ETC complex I inhibitor Metformin into our drug regimen to study the effects of co-targeting of OXPHOS and mitochondrial translation on tumor progression; additional inclusion of Paclitaxel in this scheme would permit targeting of non-CSC dividing cells within the tumor (Fig. 7b). Different drug regimens exhibited distinct tumor suppression kinetics over the treatment window (Fig. 7d–i). Among the various pharmacological interventions assessed, Metformin administered as a monotherapy or in combination with Doxycycline, Paclitaxel, or Erythromycin produced a significant inhibitory effect on tumor growth at Day 7 (Fig. 7d–i). Notably, the combination of Metformin with Erythromycin and Paclitaxel (Metformin + ery + Pax) exhibited a comparatively reduced, yet still significant, tumor-suppressive effect relative to the vehicle control (Fig. 7d–i). In contrast, the regimen comprising Metformin, Doxycycline and Paclitaxel (Metformin + Doxy + pax) did not result in any noticeable tumor reduction at Day 7 (Fig. 7d–i). However, extended administration of either Metformin + Ery + Pax or Metformin + Doxy + Pax led to tumor inhibition at Day 14, comparable to other drug regimens. Upon cession of the treatment at Day21, Metformin + Ery + Pax demonstrated the most pronounced tumor inhibitory effect among all groups evaluated (Fig. 7d–i, ii, Supplementary Fig. 7b). This differential tumor inhibitory effect shown by Metformin + Ery + Pax on Days 7 to Day 21, may suggest an initial delayed synergetic interaction, potentially due to undetermined- pharmacodynamic factors.

Altogether, our findings strongly indicate that targeting mitochondrial metabolism and translation, which are crucial for CSC self-renewal and maintenance, in conjunction with conventional chemotherapeutic agents that target the non-CSC tumor population, can enhance treatment efficacy.

## Discussion

Challenges in improving HGSC patient survival include intrinsic drug resistance, tumor heterogeneity, resilient cellular energetics and metabolic heterogeneity, all of which depend on the cellular environment [3, 27]. Cellular energetics is primarily attributed to the ability of mitochondria to respond to microenvironmental changes and altered gene and protein expression that also supports stem-like cell maintenance in a tissue-specific manner [28–37]. Serum depletion recapitulates the in-situ behavior of CSC’s and their ability to opportunistically exploit available resources within a nutrient-poor tumor microenvironment (TME; [38, 39]). Quiescent CSCs possess lower yet more efficient energy generation as their metabolism is predominantly confined to minimal maintenance levels; consequently, these cells preferentially rely on slower anaerobic respiration processes such as OXPHOS, over glycolysis [39]. As the TME becomes hypoxic and nutrient scarce, CSCs also metabolically adapt to utilize free fatty acids for survival [38]. Hence, our identification of altered mitochondrial pathways including enrichment of mitochondrial translation, FAO, TCA and OXPHOS, with metabolites like succinate, oxaloacetate, fumarate and iso-citric acid, accompanied by acquisition of stemness following serum deprivation irrespective of their intrinsic cellular plasticity, is important.

At an ultrastructural level, the above changes are also associated with orchestrating mitochondrial states between fusion and fission, along with conserved patterns of cristae remodeling across all cellular phenotypes. The ensuing cristae dynamics is directly linked with cellular metabolic flux since ETC complexes are assembled along their membranes and ATP synthase at their edges [40–42]. We thus observed increased number of leaner cristae, tight CJs, OPA1 levels which along with mitochondrial contact sites and cristae organization system (MICOS) complex can play a crucial role in efficient ATP synthesis by minimizing the proton leakage with the tight CJ openings [43]. However, neither fused nor fragmented mitochondrial morphology is universally linked to cancer stemness. 2D-TEM analysis also revealed altered inter-organelle communication between the endoplasmic reticulum (ER) and mitochondria with reduced mitochondrial-ER contact (MERC) distance and increased coverage following serum deprivation (Supplementary Fig. 8). These findings need a comprehensive investigation in the future, as MERC distance is known to impact lipid metabolism, calcium-mediated OXPHOS, and autophagy [44–47]. Various studies have used computer simulation and mathematical models to investigate mitochondrial fission-fusion dynamics and their response to different substrate inputs, very few of which have integrated the mitochondrial dynamics with cellular energetics especially, details regarding OXPHOS [48–52]. In this context, our study is distinctive and one of its kind, as it demonstrated the potential to predict cellular bioenergetics by incorporating ultrastructural details from 2D-TEM images along with expression data of the fusion-fission associated OPA1 and DRP1, which are central to mitochondrial dynamics. All observations were valid across the entire gradient of phenotypes, thereby accounting for disease heterogeneity.

The vulnerability created through increased dependency of CSCs on mitochondrial OXPHOS has led to development of new drugs (Mitoriboscins, Mitoketoscins, MitoTam) or repurposing of earlier FDA-approved drugs such as antibiotics, Metformin etc. [26, 53–56]. Ongoing clinical investigations with compounds like IACS-010759 have shown promise in leukemia and glioma models [57]. However, metabolic heterogeneity within tumor populations limits the efficacy of these treatments in several instances. Our study too demonstrates the potential of targeting mitochondrial translation and OXPHOS using antibiotics and Metformin respectively; Etomoxir also compromise these metabolic pathways through inhibition of the CPT1 transporter. The combination of these agents with conventional chemotherapy that targets the non-CSC population, significantly inhibited HGSC tumor progression in vivo.

## Materials and methods

### Cell culture

Five HGSC cell lines used in the study include, A4 (established earlier in our lab from the ascites of a HGSC patient, [58]), OVCAR3 (sourced from BRIC-NCCS Cell Repository, Pune, India), CaOV3 and OVCA420 (provided by Prof. Judith Clements, Translational Research Institute, Australia) and OVMZ6 (from Prof. Viktor Magdalen (Klinische Forschergruppe der Frauenklinik der Tu, Munchen). All cell lines were maintained at 37 °C under 5% CO_2_ in a humidified incubator, cultured in appropriate media—OVCAR3, CaOV3 and OVCA420 in RPMI 1640 (Gibco) + 10% fetal bovine serum (FBS,Gibco), A4 in Minimal Essential Medium (MEM;Gibco) + 5% FBS + 1% non-essential amino acid (Gibco), OVMZ6 in Dulbecco’s Modified Essential Medium (DMEM;Gibco) + 10% FBS + 100uM asparagine and 100uM arginine (Merck-Sigma). For serum starvation (SS) and serum-fed condition (+S), cells were allowed to grow for 24 h following which the media was replaced with equal volume of either serum-free or complete media respectively. All cell lines were authenticated via short tandem repeat (STR) profiling (Project No. STR24082023), employing GeneMapper™ ID-X Software version 1.5 for analysis. All cell lines were also confirmed to be free of mycoplasma contamination.

### Cell cycle analysis, assays for self-renewal and cell survival, PKH label chase

48 h SS or +S HGSC cells were used for cell cycle and self-renewal assays as described earlier [58, 59]. Briefly, label chase was performed with the vital lipophilic membrane dye fluorophore PKH26 (Merck, #PKH26GL), following the manufacturer’s instructions. PKH26 fluorescence intensity was assessed at designated time points (0, 24, 48, 72, and 96 h) using a BD FACS Canto flow cytometer. A4 3D-spehroid formation capability was assayed in response to IC50 concentration of different drugs as described earlier [10]. Briefly, 5000 cells were seeded in each well of a 96-well ultra-low attachment plate and cultured in minimum essential medium containing 1% serum that was replenished every 48 h along with drug/vehicle control-containing media as per individual groups/experiments for 14 days. Images were captured using Olympus FV3000.

OCT4 (forward-5’GACAACAATGAAAATCTTCAGGAGA3’, reverse 5’TTCTGGCGCCGGTTACAGAACCA3’), NANOG (forward- 5’AGTCCCAAAGGCAAACAACCCACTTC3’, reverse- 5’ATCTGCTGGAGGCTGAGGTATTTCTGTCTC3’) and SOX2 (forward- 5’TGGCGAACCATCTCTGTGGT3’, reverse- 5’CCAACGGTGTCAACCTGCAT3’) expression were profiled for self-renewal through q-PCR analysis as described earlier [59]. MTT [3-(4,5-dimethylthiazol-2-yl)-2,5-diphenyltetrazo lium bromide] (Sigma-Aldrich, #M2128) assays were used for determination of IC50 values and cell viability under glycolysis (2-deoxy-D-glucose, Sigma-Aldrich, #D8375 and sodium dichloroacetate, Sigma-Aldrich, #347795) or OXPHOS (Rotenone, Sigma-Aldrich, #R8875, Antimycin A, Sigma-Aldrich, #A8674 and Oligomycin, Sigma-Aldrich, #O4876) inhibitors, CPT1 inhibitor (Etomoxir, Sigma-Aldrich, #236020) and antibiotic drugs (Doxycycline, Sigma-Aldrich; Erythromycin, Sigma-Aldrich, #E5389; Chloramphenicol, Sigma-Aldrich; Tetracycline, Sigma-Aldrich) as described earlier [60]. All experimental data presented were obtained from triplicate experiments to ensure reproducibility.

### In solution digestion, acquisition of spectrometry profiles, label free quantification (LFQ)-based pathway analysis and data representation

HGSC cells harvested 48 h post-treatment (48h_SS) or as controls (+S) in triplicate were processed per previously established protocols [61]. Data acquisition was performed using an Orbitrap Fusion™ mass spectrometer (Thermo Fisher Scientific) coupled with an EASY-nLC™ 1200 nano-flow liquid chromatography (LC) system (Thermo Fisher Scientific), and an EASY Spray column (50 cm × 75 µm ID, PepMap C18). LFQ analysis was performed using MaxQuant version 1.6.17.0 [62, 63], followed by downstream data processing and statistical analysis in Perseus (version 1.6.14.0, [64]). A comprehensive pipeline was developed to identify differentially expressed proteins between +S and SS conditions [upregulated defined as a fold change (FC) > 2 downregulated as FC < 0.5], or exclusively expressed proteins in either condition; and were further visualized in volcano plots. Proteins identified with at least two peptides and 10% sequence coverage in at least two of the three replicates were included in the analysis. Proteins which were exclusively expressed and significantly upregulated (>2FC) in each phenotype following SS were collectively considered as SS-enriched proteins and subjected to pathway enrichment analysis, which was conducted using the REACTOME pathway database v84, Gene Set Enrichment Analysis (GSEA), Cytoscape with the ClueGO plugin, and the DAVID knowledgebase v2022q3 [65–68]. Mitochondrial-specific pathway analysis was performed using the Mitocarta 3.0 database [69]. Heatmaps were generated using MeV version 4.9.0, with Euclidean distance applied for hierarchical clustering of samples and genes/proteins.

### Metabolomics

Prechilled methanol: acetonitrile: water (1:1:0.5) solvent was added to HGSC cell line pellets (48h_SS & 48h_+S, in triplicates) to extract metabolites, followed by freeze-thaw cycles and sonication. Samples were centrifuged at 16,000 *G* for 20 min at 4 °C, the supernatant lyophilized and resuspended in 80% methanol. Liquid chromatography-high-resolution mass spectrometry (LC-HRMS) was performed on Shimadzu Prominence HPLC system (Shimadzu Corporation, Japan) connected to a SCIEX QTRAP 6500+ hybrid triple quadrupole/ion trap mass spectrometer. Samples were loaded onto a Waters Atlantis T3 column (5 µm, 4.6 × 150 mm) maintained at 40 °C. Solvents used were 0.1% formic acid in LC/MS grade water (buffer A) and 0.1% formic acid in acetonitrile (buffer B), with a gradient from 0 to 98% B over 38 min at 0.5 mL/min, followed by 5 min at 98% B and 5 min of re-equilibration and was operated in negative ion mode. Multiple reaction monitoring (MRM) was applied for TCA cycle metabolites (Merck-Sigma, #ML0010) and D2-L-phenylalanine was added as an internal control. Data were analyzed using SCIEX-OS (Version 3.0.0.3339) at the BRIC-NCCS proteomic facility.

### Glucose consumption, lactate production, ROS, mitochondrial mass analysis, TMRM assay, ATP assay and mitochondrial DNA copy number analysis

Glucose consumption and lactate production assays, and reactive oxygen species (ROS) analysis were performed as described earlier [59]. MitoTracker green FM (Cell Signaling Technology, #9074) was used (150 nM) for the mitochondrial mass (mito-mass) analysis. Mitochondrial potential differences and activity were examined using the image-iT TMRM reagent (ThermoFisher Scientific, #I34361). Briefly, HGSC cells exposed to +S and SS conditions were incubated with 100 nM TMRM reagent for 30 min in incubator, harvested, washed with PBS and fluorescence was acquired. All FACS data were acquired in BD FACS Canto and analysis were performed in FlowJo software v.6. For intracellular ATP levels, ATP assay kit (Abcam, #ab83355) was used according to manufacturer’s instruction. Colorimetric reading for the same was acquired at 570 nm using a microplate reader and readings were normalized to cell numbers. For mtDNA copy number analysis, 48 h SS vs +S HGSC cells were incubated overnight at 55 °C with digestion buffer (10 mM Tris-Cl, 100 mM NaCl, 0.5% SDS, 25 mM EDTA, 0.1 mg/ml proteinase K). DNA was separated from RNA, proteins, and debris using phenol:chloroform: isopropanol (25:24:1), followed by centrifugation. The aqueous layer was treated with sodium acetate and ethanol, centrifuged, washed with 70% ethanol, dried, and dissolved in NFW. Quantitative real-time PCR was performed with SYBR Green PCR master mix (TaKaRa, #RR820) on an Applied Biosystems StepOne Plus PCR system. Human cytochrome-b (forward- 5’GCGTCCTTGCCCTATTACTATC3’, reverse -5’CTTACTGGTTGTCCTCCGATTC3’) for mitochondrial DNA (mtDNA) and human RPL13A (forward- 5’CTCAAGGTCGTGCGTCTG3’, reverse- 5’TGGCTTTCTCTTTCCTCTTCTC3’) for nuclear DNA (nuDNA) primers were used for the analysis. Cycle threshold (Ct) values from triplicate reactions in qPCR were computed using the following equation to calculate the relative mtDNA content.

$${Delta\; Ct}\left(\Delta {Ct}\right)=\left({nuDNA\; Ct}-{mtDNAct}\right){;Relative\; mtDNA\; content}=2* ({2}^{\Delta {Ct}})$$

All experimental data presented were obtained from at least triplicate experiments to ensure reproducibility

### Immunoblotting

Immunoblotting of 48 h SS and +S HGSC samples was performed as described earlier [70]. OPA1 (CST, #80471, 1:1000) and DRP1 (CST, #8570, 1: 1000) primary antibodies were used. After incubation with secondary antibody for 2 h at room temperature, membranes were developed using SuperSignal West Pico PLUS chemiluminescent substrate (ThermoFisher Scientific, #34579). Quantitative data presented are derived from triplicate measurements.

### Lipid droplet (LD) staining and confocal mitochondrial network analysis

48 h +S or SS HGSC (each in triplicate) cells were fixed with 2% paraformaldehyde for 10 min, stained with 2 µM BODIPY^TM^ 493/503 (ThermoFisher Scientific, #D3922,) for 30 min and Hoechst for 10 min in the dark. For mitochondrial network analysis, 72 h HGSC + S and SS cells were fixed and stained with 100 nM MitoTracker deep red for 30 min followed by Hoechst staining. Images were captured on an Olympus FV3000 and analyzed with ImageJ (V1.54 f). Mitochondrial network analysis was performed using the “MitochondrialAnalyzer” plugin (V2.1.0, [71]).

### Transmission electron microscopy (TEM)

Cells from 100 mm plates were pelleted down and washed with cold PBS followed by fixation using 3% glutaraldehyde for 2 h at 4 °C. After washing with 0.1 M sodium cacodylate buffer, cells were fixed using a second fixative, 1% osmium tetroxide for 1 h at 4 °C in dark. After dehydration and resin infiltration, cells were embedded in Araldite B resin. Further, ultrathin sections with 70 nm thickness were cut on Leica UC7 ultra-microtome and collected on copper 200 mesh grids. Cells were stained with uranyl acetate and lead citrate, followed by scanning using JEOL JEM 1400 PLUS transmission electron microscope at 120 kV. Images were acquired using EMSIS TENGRA camera. All the TEM image acquisition and sample processing were carried out in electron microscope facility, ACTREC, Mumbai. Quantitative data represented were derived from at least 15 TEM images from each +S and SS derivatives of HGSC phenotypes

### Mathematical modeling

Mathematical modeling was performed in Google Colaboratory (https://colab.research.google.com/, [72]) with Python V3.10, the following abbreviations were used in differential equations.

$${N}_{{HU}}{isthenumberofunfusedHealthymitochondria}$$

$${N}_{{DU}}{isthenumberofunfuseddeviantmitochondria}$$

$${N}_{{HF}}{isthenumberoffusedHealthymitochondria}$$

$${N}_{{DF}}{isthenumberoffuseddefectivemitochondria}$$

$${M}_{H}{isthespecificmitophagyrateofhealthymitochondria}$$

$${M}_{D}{isthespecificmitophagyrateofdefectivemitochondria}$$

$$\theta {istheATPproductionfactor}$$

$${c}_{n}{isthenumberofcristaeperunitlength}$$

$$\varPsi {isthetransmembranepotential}$$

$${c}_{w}{isthecristaewidth}$$

Codes used for the mathematical model studies can be provided on request.

### Xenograft generation and drug evaluation

All procedures were performed on approval from the Institutional Animal Ethics Committee (IAEC, project no. B-388), and mice were bred and maintained at the BRIC-NCCS Experimental Animal Facility. Subcutaneous xenografts were established by injecting 2.5 × 10^6^ A4 cells into 6–8-week-old female NOD/SCID mice; wherever described some were pre-labeled with PKH26. Mice were randomized to different groups (*n* = at least 4 per group) and treatment initiated on Day 14 post-cell injection. The following doses were administered intraperitoneally: 25 mg/kg of Paclitaxel (Sigma-Aldrich, #T7191), 50 mg/kg of each Doxycycline and Erythromycin (Sigma-Aldrich, #E5389), and 250 mg/kg of Metformin (Sigma-Aldrich, #317240); detailed description of drug regimens is provided in Fig. 7b (for the metformin included mice experiments 3.5 × 10^6^ cells were inoculated for the subcutaneous xenograft generation). In vivo tumor progression was monitored and measured on Days 7th, 14th, and 21st following treatment by measuring tumor volume using the formula: 0.5 × length × (width)^2^. No blinding was performed while assessing the tumor measurements. Mice were euthanized on completion of the drug regimen (Day 21) and tumors were harvested. A “tumor inhibitory score” of each drug regimen was computed based on differences in % of PKH^hi^ and PKH^low^ fractions (i.e CSC and progenitor populations) normalized to tumor volumes between treated and vehicle control tumors.

$${Tumorinhibitoryscore}={Control}\left(\frac{ \% {PKHhi}+ \% {PKHlow}}{C.{tumorvolume}}\right)-\,Test\left(\frac{ \% {PKHhi}+ \% {PKHlow}}{T.{tumorvolume}}\right)$$

- *C. tumor:* *Vehicle control tumor*
- *T. tumor:* *Test* (*different drugs*) *tumor*
- *PKH*^*hi*^ *:* *CSC* *fractions*
- *PKH*^*low*^*:* *Progenitors*

### Statistics

Unless specified otherwise, all experiments were conducted with a minimum of three independent replicates. Statistical comparisons between two groups were performed with two-tailed Student’s *t* test, while comparisons involving three or more groups were analyzed using one-way analysis of variance (ANOVA). Data are presented as the mean ± standard error of the mean (SEM), unless otherwise noted. Statistical significance was determined using *p*-values, with *p < 0.05, **p < 0.01, and ***p < 0.001. Graphical representations and statistical analyses were performed using GraphPad Prism software (version 8.4.2, [73]).

## Supplementary information

Supplementary Figures

Supplementary Table 1

Western Blot Supplementary File

## Data Availability

The data that support the findings of this study are available within the article and its Supplementary files. Proteomic data generated through mass spectrometry are submitted to the ProteomeXchange Consortium via the PRIDE repository [74], under the dataset identifier PXD062888.
