# Supplementary material for: Targeting mitochondrial translation and OXPHOS in high-grade serous ovarian carcinoma eliminates stem-like cells
Source: Cell Death Dis. 2025 Oct 6;16(1):676. doi: 10.1038/s41419-025-07987-1 (PMC12501233; doi:10.1038/s41419-025-07987-1)
Supplement: Supplementary file 1 — Supplementary Figures [file 41419_2025_7987_MOESM1_ESM.pdf]

Supplementary Fig. 1

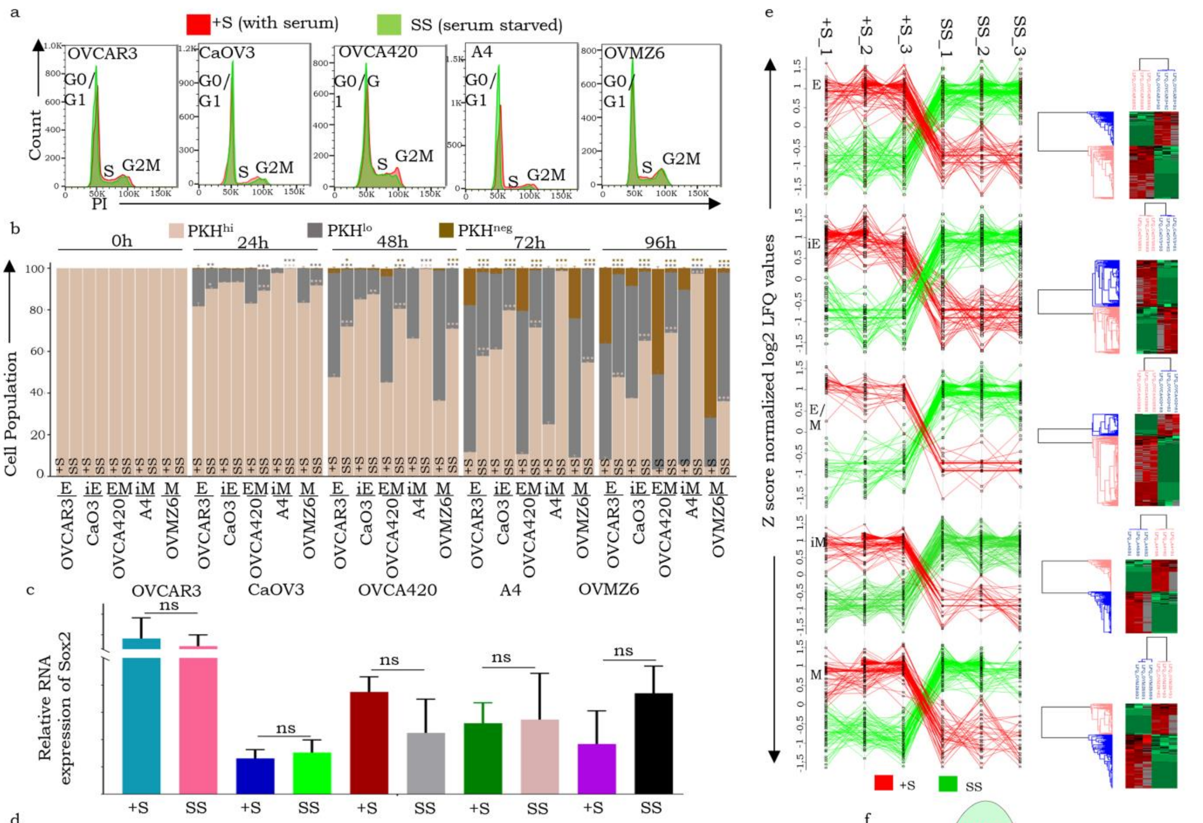


d

| Classes | (E) | (iE) | (E/M) | (iM) | (M) |
|---|---|---|---|---|---|
| +S (exclusive) | 92 | 87 | 38 | 82 | 114 |
| +S (upregulated) | 38 | 28 | 5 | 17 | 24 |
| SS (exclusive) | 147 | 96 | 117 | 118 | 111 |
| SS (upregulated) | 60 | 30 | 30 | 64 | 61 |

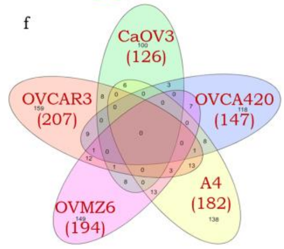


***Supplementary Fig.1**. **a.** Representative histogram indicating cell cycling of HGSC cells grown under SS (96h) and +S conditions (green-SS, red-+S); **b.** Stacked-bar graph indicating the PKH$^{hi}$ PKH$^{lo}$ and PKH$^{neg}$ fractions in HGSC +S and SS derivatives at different time interval (0h, 24h, 48h, 72h and 96h); **c**. Bar graph indicating relative levels of Sox2 expression in HGSC phenotypes under +S and SS conditions; **d**. Table consolidating the number of upregulated and exclusive proteins under SS and +S conditions across the phenotypic spectrum (E- Epithelial-OVCAR3, iE-intermediate Epithelial-CAOV3, E/M-Epithelial/Mesenchymal hybrid-OVCA420, iM- intermediate Mesenchymal-A4, M-Mesenchymal-OVMZ6); **e**. Left panel- profile plot representing SS and +S enriched proteins, right panel- heat map of the same; **f**. Venn-diagram indicating shared and exclusive proteins between HGSC phenotypes under conditions of serum starvation (SS).* **p < 0.05, **p < 0.01 and ***p < 0.001.

Supplementary Fig.2

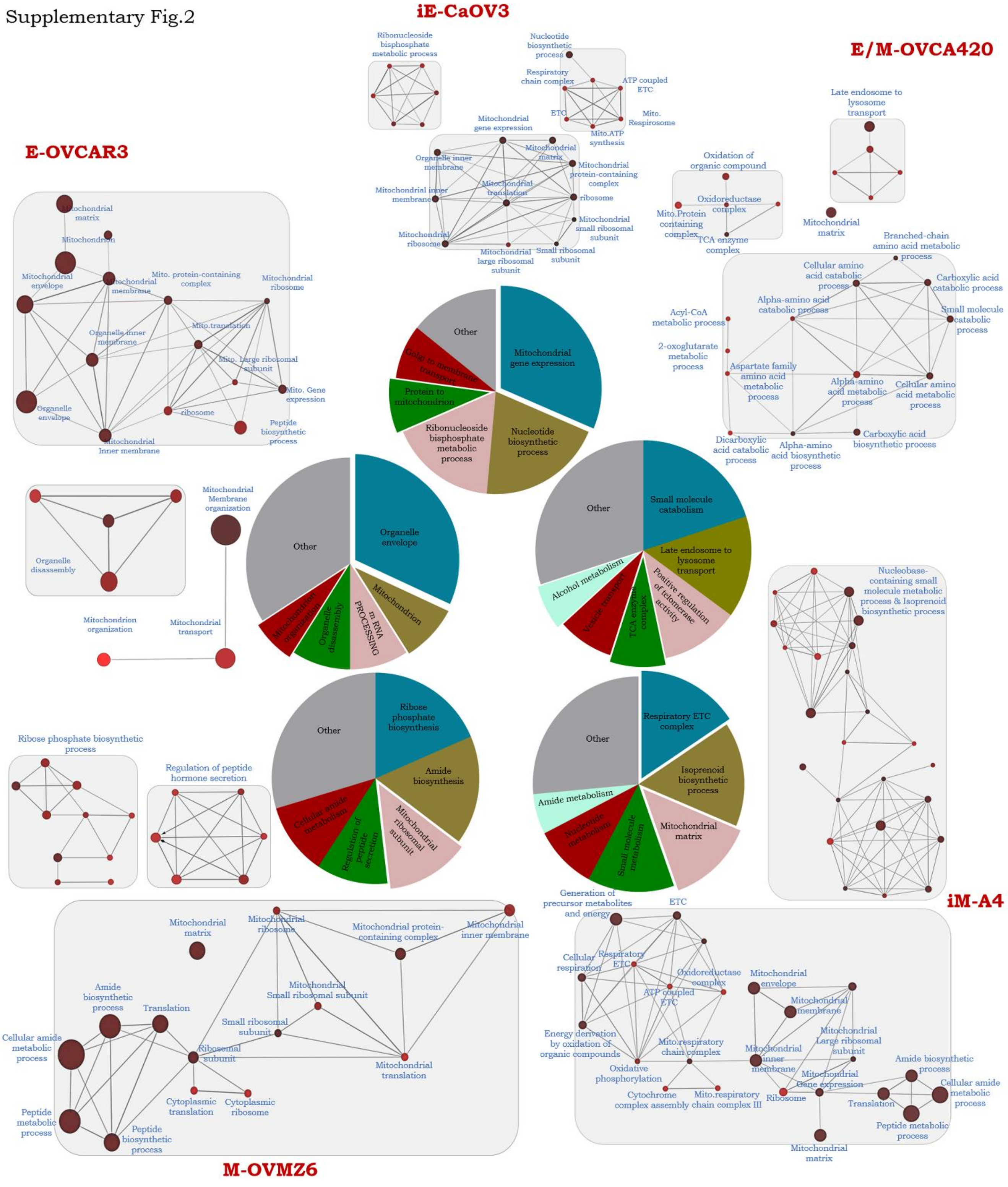


***Supplementary Fig. 2***. *Representative cluster plots and pie diagram indicating enriched GO biological process and cellular components in HSGC phenotypes following SS conditions. Regardless of the cellular phenotype, serum starvation response engages different mitochondrial-related proteins*

Supplementary Fig. 3.

a-i a-ii

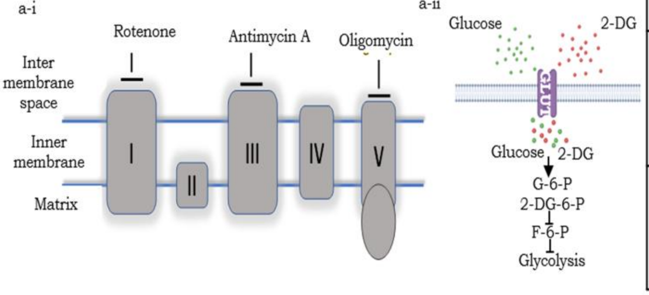


b

| Class | Drugs | OVCAR3 | CaOV3 | OVCA420 | A4 | OVMZ6 |
|---|---|---|---|---|---|---|
| OXPHOS inhibitors | Oligomycin | 22 | 13 | 14.5 | 13 | 19 |
| | Rotenone | 18.5 | 11 | 22 | 14 | 11 |
| | Antimycin A | 40 | 13 | 18 | 22 | 11.2 |
| Glycolysis inhibitors | 2-deoxy-d-glucose | 338 | 153 | 340 | 264 | 250 |
| | Sodium di-chloroacetate | 19.2 | 32 | 25 | 28 | 24 |

c

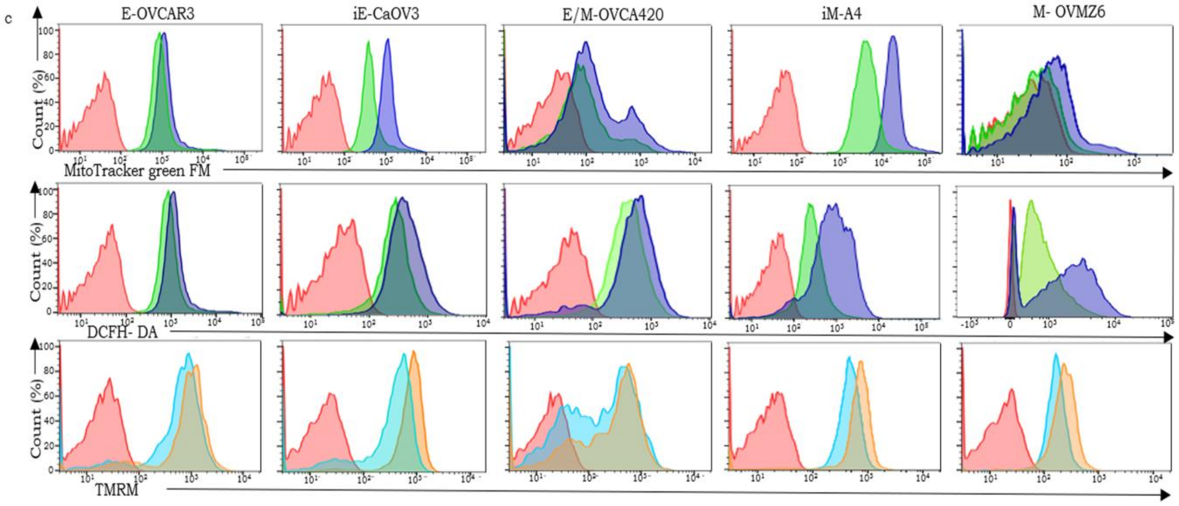


***Supplementary Fig. 3***. *HGSC cells prefer OXPHOS under SS accompanied by significantly increased active mitochondria.* ***a-i, a-ii***. *Schematic indicating site of action of OXPHOS and glycolysis inhibitors respectively;* ***b***. *IC50 values of the specific inhibitors on HGSC cell lines;* ***c***. *Histograms representing, mitochondrial mass (MitoTracker green FM-upper panel), ROS (DCFH-DA-middle panel) and mitochondrial potential/ activity (TMRM- lower panel) at 72h under SS & +S conditions. Violet and green colour in the upper and middle panels represent SS and +S histogram respectively, while the same is represented by orange and blue, respectively, in the lower TMRM panel.*

Supplementary Fig.4.

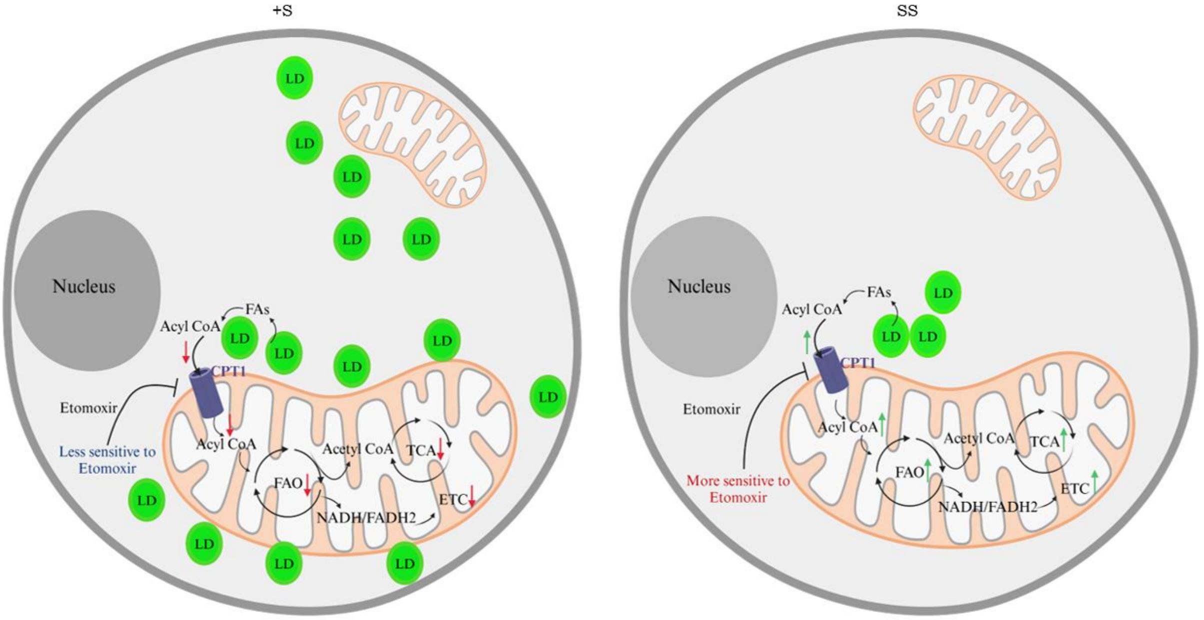


Lipolysis↓
No: of Lipid droplets↑
Sensitivity to Etomoxir↓
FAO↓
TCA ↓

Lipolysis↑
No: of Lipid droplets ↓
Sensitivity to Etomoxir↑
FAO↑
TCA ↑

***Supplementary Fig. 4.*** *HGSCs utilize the stored fatty acids to fuel TCA and subsequently OXPHOS after serum deprivation*

Supplementary Fig.5

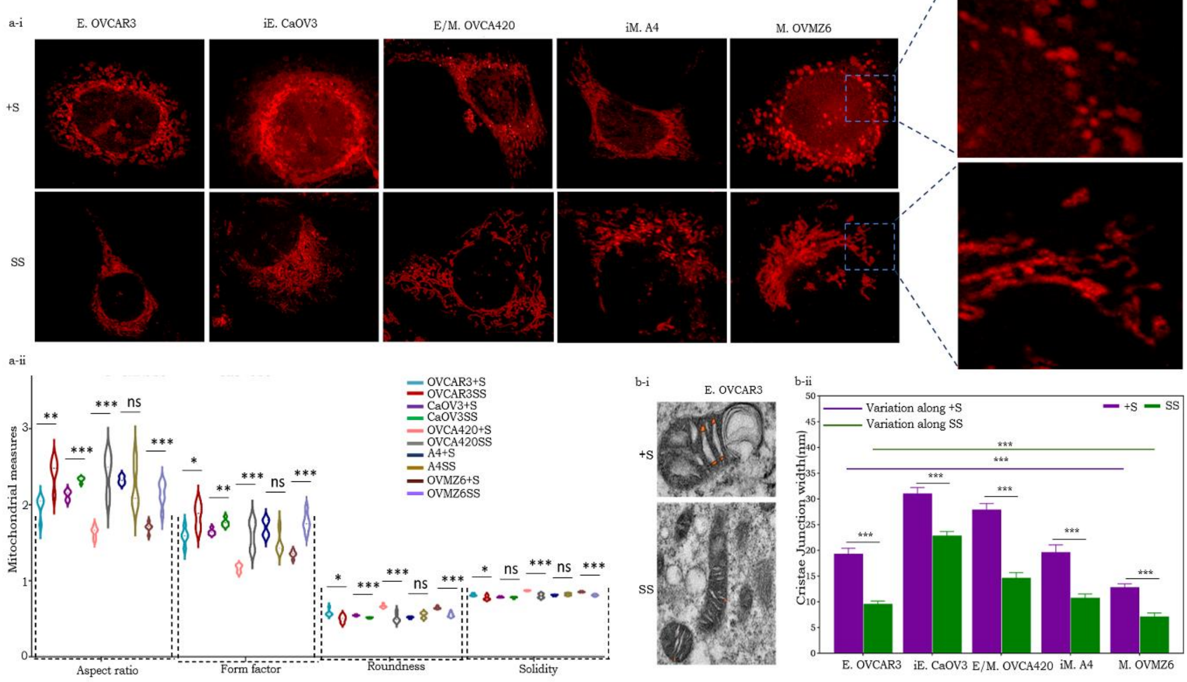


***Supplementary Fig. 5**. Under SS conditions, HGSC cells mitochondria undergo fusion to fulfil the energy demands necessary for their survival: **a-i**. MitoTracker deep red confocal microscopy images indicating the mitochondrial morphology across HGSC phenotypes in the presence (upper panel) and absence (lower panel) of serum; **a-ii**. Violin plot representing the mitochondrial structural parameters analysed on ImageJ on 48h SS vs +S samples (derived from at least 50 individual cells per condition across the various phenotypes); **b-i.** Representative confocal images highlighting CJs (orange in colour) in 48h +S and SS OVCAR3 cells; **b-ii**. Bar-graph indicating CI width in HGSC cells under SS and +S conditions.* **p < 0.05, **p < 0.01 and ***p < 0.001.

Supplementary Fig.6

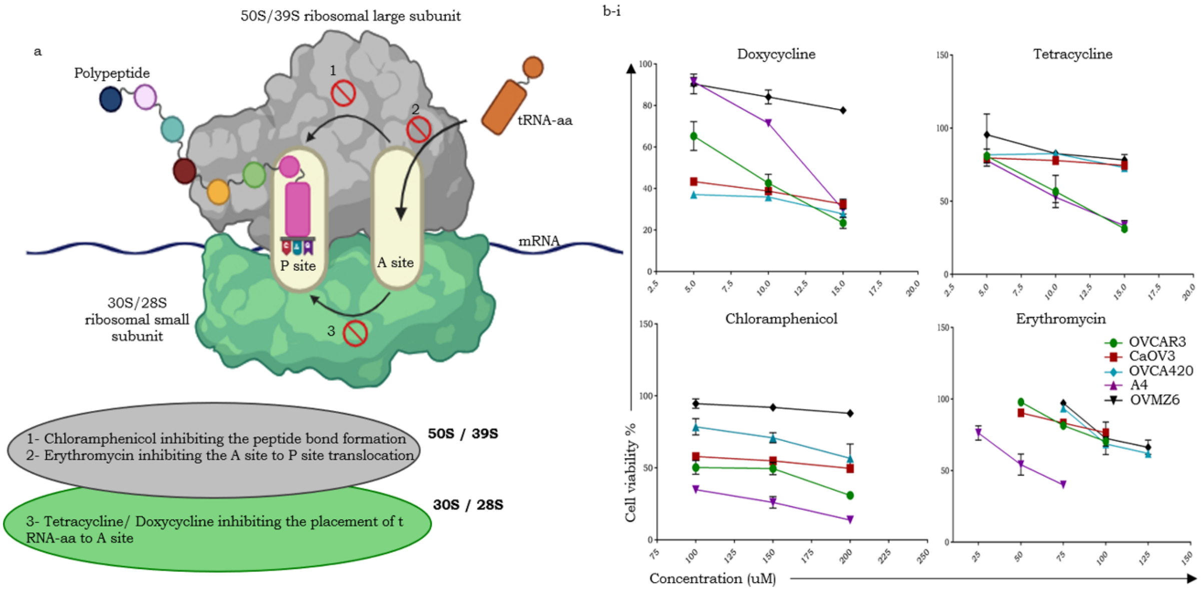


b-ii

| Classes | Drugs | OVCAR3 | CaOV3 | OVCA420 | A4 | OVMZ6 |
|---|---|---|---|---|---|---|
| Antibiotic | Chloramphenicol (uM) | 174 | 290 | 312 | 77 | 700 |
| | Tetracycline (uM) | 12 | 25 | 30 | 12 | 23 |
| | Erythromycin (uM) | 134 | 190 | 153 | 62 | 150 |
| | Doxycycline (uM) | 18 | 10 | 4.8 | 23 | 36 |

***Supplementary Fig. 6.*** *Site and mechanism of action of antibiotics and their IC50 values in different HGSC phenotypes.* ***a.*** *Schematic representation of site and mode of action of different classes of antibiotics;* ***b-i.*** *Line plots representing IC50 value determination of antibiotics in different HGSC phenotypes;* ***b-ii****. Table indicating the computed IC50 values of antibiotics in different HGSC cell lines.*

Supplementary Fig.7.

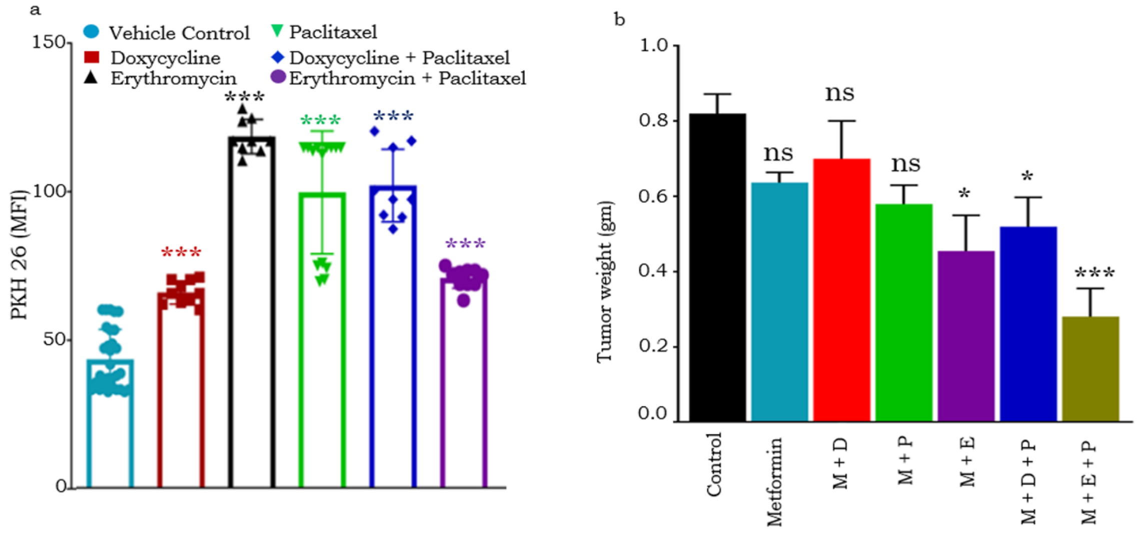


***Supplementary Fig. 7**. Tumor inhibitory effects of OXPHOS and mitochondrial translation inhibitors.* ***a**. Bar graph indicating PKH quenching in A4 tumors harvested at Day 21 of different drug regimens;* ***b**. Bar-graph indicating A4 tumor weights at Day 21 of different drug regimens.* **$p < 0.05$, **$p < 0.01$ and ***$p < 0.001$.

Supplementary Fig.8

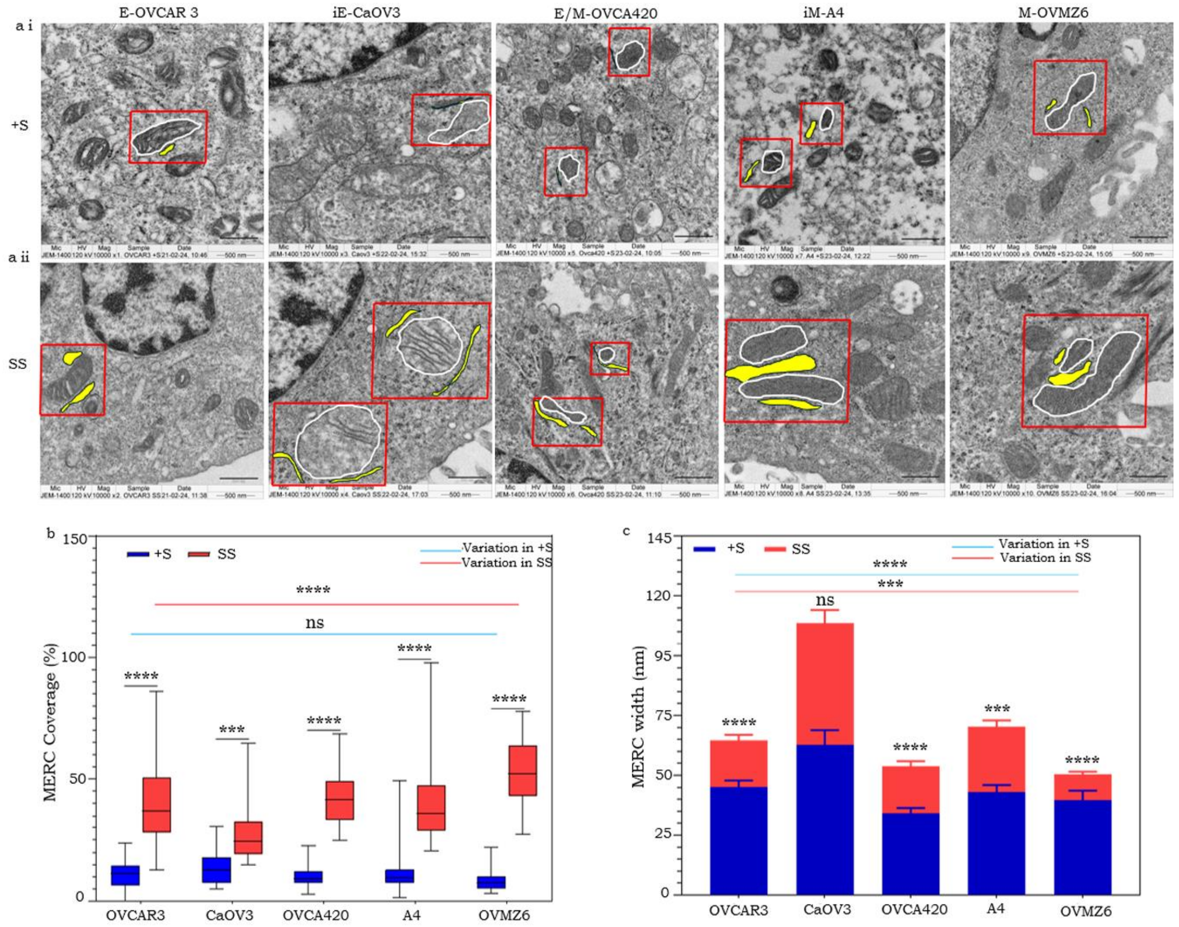


***Supplementary Fig. 8**. Serum starvation led to reduced mitochondrial-endoplasmic-reticulum contact (MERC) distance across all phenotypes. **a**. Representative 2D-TEM images highlighting the MERC distance under +S (upper panel) and SS (lower panel) conditions; **b**. Floating bar-graphs indicating the MERC coverage in HGSC phenotypes under +S and SS conditions; **c**. Stacked bar-graph representing the MERC width across the phenotypes under +S (blue) and SS (red) conditions.* *p < 0.05, **p < 0.01, ***p < 0.001 and ****p < 0.0001
