# Supplementary figures and images for: Targeting mitochondrial translation and OXPHOS in high-grade serous ovarian carcinoma eliminates stem-like cells
Source: Cell Death Dis. 2025 Oct 6;16(1):676. doi: 10.1038/s41419-025-07987-1 (PMC12501233; doi:10.1038/s41419-025-07987-1)

# Western Blot\_ Supplementary File

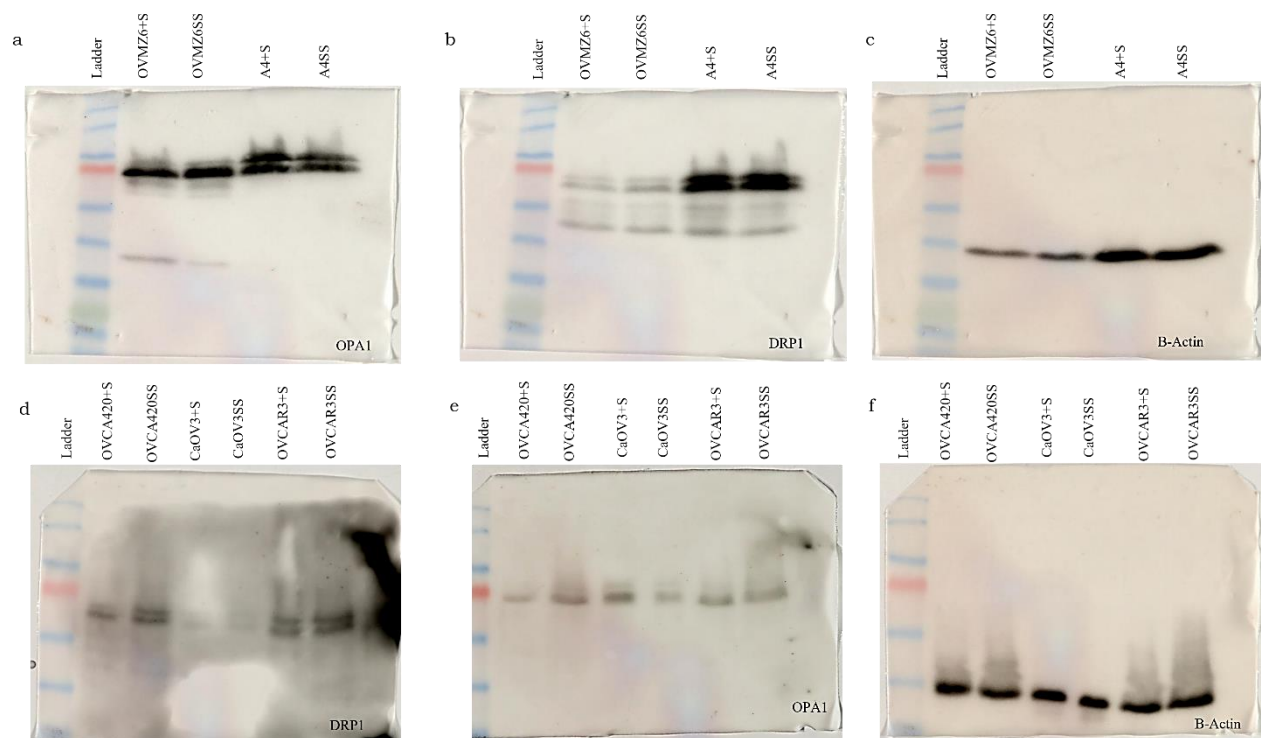

Supplement: Supplementary file 3 — Western Blot Supplementary File [file 41419_2025_7987_MOESM3_ESM.pdf]
